# Natural Product Toosendanin Suppresses the Malignant Development of Skin Melanoma by Targeting BNC2 for Degradation

**DOI:** 10.1002/advs.77458

**Published:** 2026-08-30

**Authors:** Hui Dai, Xin Mei Zhang, Jing Cheng Zhang, Xu Ting Deng, Qiao Liu, Mustafa Hussein Ali, Fang Zhou Yin, Wu Yin

**Affiliations:** ^1^ State Key lab of Pharmaceutical Biotechnology (SKLPB) College of Life Sciences in Nanjing University (Xianlin Campus) Nanjing University Nanjing China; ^2^ Nanjing First Hospital Nanjing Medical University Nanjing China; ^3^ School of Pharmacy Nanjing University of Chinese Medicine Nanjing China

**Keywords:** BNC2, metastasis, PIK3CA, proliferation, SKCM, TSN

## Abstract

Toosendanin (TSN) is a highly potent natural anticancer compound derived from the fruit of Fructus Meliae Toosendan. In this study, TSN inhibited melanoma cell proliferation and cell‐cycle progression, induced apoptosis, and suppressed metastasis in both in vitro and in vivo models. TSN is a potent PI3K inhibitor; here, we identify basonuclin 2 (BNC2) as a transcription factor critically involved in TSN's suppression of *PIK3CA* transcription in melanoma cells. In clinical samples and melanoma datasets, BNC2 expression is substantially elevated in skin cutaneous melanoma and correlates with poor patient metastasis. Functional studies demonstrate that BNC2 promotes melanoma cell proliferation and migration. TSN directly binds to BNC2 and CRBN, the substrate receptor subunit of the E3 ubiquitin ligase complex, acting as a molecular glue to induce ubiquitin‐dependent degradation of BNC2 and subsequent downregulation of *PIK3CA* transcription. TSN nanoliposome encapsulation improves cellular uptake, tumor targeting, and anti‐metastatic efficacy of TSN in vivo. These findings reveal a context‐dependent oncogenic function of BNC2 in melanoma, contrasting with its previously described tumor‐suppressive roles in other cancers, and establish TSN as a small‐molecule degrader of BNC2 with therapeutic potential for melanoma.

## Graphical abstract

BNC2 exhibits context‐dependent opposing functions across multiple cancer types. This study reveals BNC2 as an oncogenic driver of melanoma proliferation and metastasis through transcriptional activation of *PIK3CA*. The natural compound TSN simultaneously degrades BNC2 and its oncogenic partner SMAD3 via CRBN‐dependent ubiquitination. TSN nanoliposomes overcome the poor cellular uptake of free TSN, offering a promising therapeutic strategy for BNC2‐driven malignancies, including melanoma.

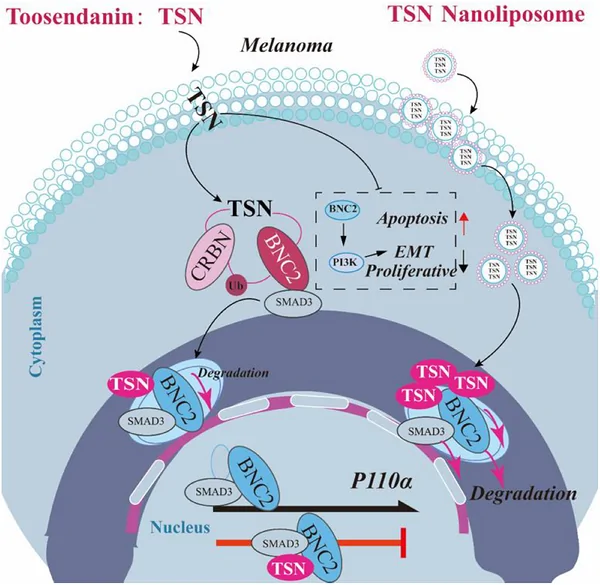

## 1 Introduction

Skin cutaneous melanoma (SKCM) is a malignant tumor arising from melanocytes, most commonly occurring in the skin, but it can also develop in the eyes and mucous membranes [1]. It represents the most aggressive and lethal form of skin cancer. Over the past few decades, the global incidence of melanoma has increased markedly, particularly among Caucasian populations [2]. According to the World Health Organisation (WHO), approximately 300 000 new cases of melanoma are diagnosed worldwide each year [3].

The primary treatment for melanoma is surgical excision of the tumor along with a margin of surrounding healthy tissue to ensure complete removal. For patients with intermediate or advanced melanoma, surgery is often combined with systemic therapies, including immune checkpoint inhibitors targeting PD‐1 (pembrolizumab and nivolumab) and CTLA‐4 (ipilimumab), radiotherapy, chemotherapy agents such as dacarbazine, carboplatin, and temozolomide, as well as targeted therapies against the MAPK pathway (vemurafenib, dabrafenib, and trametinib) [4, 5]. Despite the progress in immunotherapy and targeted therapy, first‐line treatment for melanoma still primarily relies on broad‐spectrum small‐molecule chemotherapeutic agents, either used alone or in combination with immunomodulatory drugs [6]. The treatment of advanced and metastatic melanoma remains highly challenging.

Basonuclin is a cell‐type‐specific zinc finger protein, primarily comprising basonuclin 1 (BNC1) and basonuclin 2 (BNC2). The BNC2 gene is located on mouse chromosome 4 and human chromosome 9 [7, 8]. Structurally, BNC2 contains three pairs of zinc fingers, a nuclear localization signal, and a serine‐rich domain. The extreme conservation of the BNC2 amino acid sequence suggests a critical functional role, potentially as a regulatory protein involved in DNA transcription. BNC2 is widely expressed across multiple tissues, including skin, testis, kidney, uterus, and intestine [9]. Mutations in BNC2 have been linked to a range of human disorders, including inherited skin diseases and developmental abnormalities such as adolescent idiopathic scoliosis and congenital lower urinary tract obstruction [10, 11].

The functions of BNC2 in cancer are complex and vary significantly depending on the cellular and tissue context. BNC2 has been functionally characterized as a tumor suppressor in ovarian cancer, where it participates in DNA damage repair, maintains genomic stability, and transcriptionally represses oncogenic targets [12]. In breast cancer, however, BNC2 overexpression has been linked to more aggressive tumor phenotypes, and elevated BNC2 transcript levels correlate with poor prognosis in lung and prostate cancers [13, 14, 15], suggesting that BNC2 may also possess oncogenic capacity in certain cellular milieus. The underlying mechanism of this apparent duality is likely related to BNC2's function as a transcription factor: BNC2 can form a transcriptional complex with various cofactors, such as SMAD3, to regulate distinct downstream gene expression profiles [16]. Thus, whether BNC2 acts as a tumor promoter or suppressor depends on the specific co‐regulatory network present in a particular tissue.

In addition to its association with certain malignancies, BNC2 has also been closely linked to skin pigmentation [17, 18]. BNC2 is also implicated in the pathogenesis of cutaneous squamous cell carcinoma [19], highlighting its particular relevance to skin biology. Although there are associations, the role of BNC2 in melanoma remains largely unexplored. It is still unclear whether BNC2 promotes or suppresses melanoma development and which downstream effectors are involved.

Toosendanin (TSN) is a tetracyclic triterpenoid extracted from the bark or seeds of Fructus Meliae Toosendan [20]. Accumulating evidence indicates that TSN exhibits potent anticancer activity. Our previous studies demonstrated that TSN can reverse adriamycin resistance in human breast cancer cells and suppress the expression of p110α, the protein encoded by *PIK3CA* [21]. Moreover, TSN has been shown to counteract macrophage‐mediated immunosuppression and overcome glioblastoma resistance to immunotherapy [22]. TSN also effectively inhibits the progression of several malignancies, including gastric cancer [23], prostate cancer [24], and lung cancer [25, 26].

In the present study, we found that TSN effectively suppresses both proliferation and metastasis of melanoma. Mechanistically, TSN enters the cytoplasm to induce degradation of BNC2 by CRBN‐mediated ubiquitination. BNC2 is a key transcription factor regulating *PIK3CA* transcription. By downregulating BNC2, TSN inhibits the activation of the *PIK3CA* signaling pathway in melanoma, thereby suppressing tumor metastasis and growth. Furthermore, we addressed the limited cellular uptake of TSN by formulating TSN nanoliposomes, which significantly enhanced its inhibitory effect on melanoma metastasis, while maintaining a good safety profile.

## 2 Materials and Methods

### 2.1 Materials

Trizol (19201ES60) and Hieff UNICON advanced qPCR SYBR master mix (11185ES08) were purchased from Yeasen Biotech (Beijing, China). HifairIII first strand cDNA synthesis supermix (12958ES24) for qPCR, Annexin V‐FITC/PI apoptosis detection kit (40302ES20), hieff trans liposomal transfection reagent (40802ES01), and BCA protein quantification kit (20201ES76) were purchased from Yeasen Biotech (Shanghai, China). Cell cycle and apoptosis analysis kit (C1052), nuclear and cytoplasmic protein extraction kit (P0027), dual luciferase reporter gene assay kit (RG027), and MTT cell proliferation and cytotoxicity assay kit (C0009S) were purchased from Beyotime Institute of Biotechnology (Wuhan, China). Lipofectamine 2000 transfection reagent (11668500) was purchased from Invitrogen (Carlsbad, CA, USA). Toosendanin (B20201) was acquired from Shyuanye Biotechnology (Shanghai, China). SPARKeasy endofree maxi plasmid kit (AD0306) and SDS‐PAGE protein sampling buffer (EC0013‐A) were purchased from Sparkjade (Shandong, China). Acetonitrile (LC‐MS grade) and methanol (LC‐MS grade) were purchased from Merck (Darmstadt, Germany). DMSO was purchased from Aladdin (Shanghai, China). Chip‐Kit (BK0045‐01) was purchased from ACE (Changzhou, China). DiR (HY‐D1048) was purchased from MCE (Shanghai, China).

The antibodies used in the study were as follows: Tubulin (Cat#MB0009), GAPDH (Cat#BS65483M), and Smad3 (Cat#MB65975), purchased from BIOGOT Technology (Nanjing, China). Lamin‐B1 (Cat#12987‐1‐AP), BNC2 (Cat#55220‐1‐AP), PI3K (Cat#67071‐1‐Ig), E‐cadherin (Cat#31515‐1‐AP), ZO‐1 (Cat#21773‐1‐AP), Occludin (Cat# 27260‐1‐AP), S‐100(Cat#15146‐1‐AP), and Ki67 (Cat#27309‐1‐AP) were purchased from Proteintech (Wuhan, China). BNC2 (Cat#HPA018525) was purchased from Sigma‐Aldrich (Shanghai, China).

### 2.2 Cell Lines and Cell Culture

B16‐F10 (skin melanoma cell line from C57BL/6J mice) and 293T (293T cells are modified from classical HEK293 cells) cell lines were obtained from the ATCC Cell Bank (Shanghai, China). B16‐F10 cells were cultured in RPMI‐1640 medium (BC‐M‐017), while 293T cells were grown in DMEM (BC‐M‐038), both supplemented with 10% fetal bovine serum (FBS) and 1% penicillin‐streptomycin. All cells were kept at 37°C in a humidified atmosphere with 5% CO_2_.

### 2.3 Cell Viability Assays

Cell viability was assessed using the MTT assay. Cells were seeded into 96‐well plates at a density of 5 × 10^3^ to 1 × 10^4^ cells per well. After incubation for the indicated time or treatment with the designated compounds, 20 µL of MTT solution (5 mg/mL) was added to each well, followed by incubation for an additional 2–4 h at 37°C. Subsequently, 150 µL of dimethyl sulfoxide was added to dissolve the formazan crystals, and the plate was shaken gently for 10 min to ensure complete dissolution. Absorbance was measured at 490 nm using a microplate reader.

All cell experiments were conducted with a strict solvent (DMSO) control group, and the amount of DMSO used did not exceed 1‰.

### 2.4 Colony‐Forming Assay

For the colony formation assay, cells were seeded into 6–well plates at a density of 500–1000 cells per well and cultured for 5–14 days until visible colonies formed. Cells were gently washed twice with PBS and fixed with 4% paraformaldehyde for 15 min at room temperature. After fixation, the colonies were stained with 0.1% crystal violet solution for 20–30 min, rinsed gently with tap water to remove excess dye, and air‐dried completely. The colonies were then imaged and counted under a microscope to assess cell proliferative ability.

### 2.5 Cell Apoptosis Detection

Apoptosis was assessed using an Annexin V‐FITC/PI double‐staining kit according to the manufacturer's instructions. Briefly, collected cells were washed twice with PBS and resuspended in Annexin V binding buffer at a concentration of 1 × 10^6^ cells/mL. Then, 5 µL of Annexin V‐FITC was added to 100 µL of the cell suspension and incubated at room temperature for 10–15 min in the dark. Subsequently, 5 µL of propidium iodide (PI) staining solution was added, gently mixed, and incubated for an additional 5 min in the dark. Finally, apoptosis and necrosis were analyzed by flow cytometry with an excitation wavelength of 488 nm.

### 2.6 Cell Cycle Assays

For cell cycle analysis, 1 × 10^6^ cells were collected and washed twice with PBS. The cells were fixed in 70% pre‐cooled ethanol and stored at −20°C for at least 2 h. After fixation, the cells were washed twice with PBS and resuspended in 500 µL of PI staining solution containing RNase A (50 µg/mL). The samples were incubated at 37°C for 30 min in the dark. Subsequently, the PI fluorescence intensity was detected by flow cytometry at an excitation wavelength of 488 nm, and the DNA content was analyzed to determine the percentage of cells in the G1, S, and G2/M phases.

### 2.7 Transwell Assay

Cells were seeded at a density of 2 × 10^4^ cells per well in 200 µL serum‐free DMEM medium (Corning, Tewksbury, MA, USA). The lower chamber was seeded with 600–800 µL of DMEM supplemented with 10% FBS. After incubation for 24 h, the cells were fixed with 4% paraformaldehyde for 15 min and stained with 1% crystal violet for 15–20 min at room temperature. Finally, images were taken under a fluorescence microscope.

### 2.8 RNA Extraction and Transcriptomic Analysis

B16 cells were seeded in 6‐well plates and treated with 200 nM TSN for 36 h; control cells received an equivalent volume of vehicle. Total RNA was extracted using TRIzol reagent according to the manufacturer's instructions. RNA integrity was assessed by agarose gel electrophoresis and spectrophotometry. RNA‐seq library preparation and sequencing were performed by Meiji Biotechnology Co., Ltd. on the Illumina NovaSeq X Plus platform. Differential expression analysis was conducted with the R package DESeq2. Differentially expressed genes (DEGs) were identified using the criteria of |log_2_(fold change)| > 1 and adjusted p < 0.05. KEGG pathway enrichment analysis was performed on the DEGs using the R package clusterProfiler. Each group consisted of three biological replicates (n = 3).

### 2.9 Chromatin Immunoprecipitation (ChIP)‐qPCR

B16 cells were seeded in 10 cm dishes and transfected with BNC2‐Flag overexpression plasmid. Cells were harvested 48 h post‐transfection. ChIP assays were performed using the ACE ChIP kit according to the manufacturer's instructions. Cells were crosslinked with 1% formaldehyde at room temperature for 10 min, and the reaction was quenched with glycine. After washing with PBS, cells were collected, and chromatin was sonicated to 200–500 bp. Equal amounts of chromatin were incubated overnight at 4°C with either anti‐FLAG antibody (FLAG‐IP group) or Normal IgG (IgG group), followed by incubation with Protein A/G magnetic beads at 4°C for 2 h. The beads were sequentially washed, and the bound chromatin was eluted, reverse‐crosslinked, and purified. An aliquot of 1% chromatin was reserved as Input. Purified DNA was analyzed by qPCR using SYBR Green. The target primer sequences were F: 5′‐AGTAGCACATATTGTTACCCTATTTG‐3′, R: 5′‐CAGGGAGTGGTGTTGTCATCCTAG‐3′; and the negative control primer sequences were F: 5′‐GGTACCAAGAGGAACAGATCCA‐3′, R: 5′‐CAGCAGTGATTAGAAGTCTGAGA‐3′. Each sample was run in triplicate. ChIP enrichment was quantified using the % Input method.

### 2.10 Plasmid Construction

To construct the BNC2 overexpression plasmid (pSL4‐BNC2‐Puro‐Flag), the coding sequence was amplified from B16‐F10 cDNA and cloned as a BamHI‐NheI fragment into the corresponding sites of the pSL4‐Puro mammalian expression vector (General Biology Co., Ltd., Chuzhou, China). Human BNC2 and mouse Bnc2 are highly homologous, with almost identical CDS coding regions. pCMV‐MCS‐EGFP‐BNC2‐Neo plasmids were purchased from Miaoling Biology (Wuhan, China). pCMV‐MCS‐EGFP‐Crbn‐Neo plasmid and pCMV‐MCS‐EGFP‐Crbn (TRP 383‐MUT)‐Neo plasmid were purchased from Miaoling Biology (Wuhan, China). For the construction of the luciferase reporter plasmid containing the *PIK3CA* promoter, the full‐length promoter region was PCR‐amplified, digested with KpnI and XhoI, and inserted into the pGL3‐Basic vector (Miaoling Biology, Wuhan, China). The primer sequences used for the truncated promoter constructs are listed in Table S2. pGL3‐*PIK3CA*‐promoter mutant plasmids were designed and synthesized by General Biology (Anhui, China) using overlap‐extension PCR for site‐directed mutagenesis. Successful point mutations were verified by Sanger sequencing. pEnCMV‐Smad3‐HA (mouse) and pCMV‐His‐Ub plasmids were all purchased from Miaoling Biology (Wuhan, China), and their sequences were verified by Sanger sequencing prior to use.

### 2.11 Cell Transfection

Small interfering RNAs (siRNAs) targeting BNC2/Bnc2 and Crbn were used with sequences as follows: BNC2/Bnc2 siRNA#1:5’‐GGACUUUCAUUGAGAGCAATT‐3’and Crbn siRNA#1: 5’‐UGAAAUAAGUCCAGACAAATT‐3’. A non‐specific control siRNA (negative control): 5’‐ UUCUCCGAACGUGUCACGUTT‐3’. Cell transfection with siRNAs or plasmids was performed using Lipofectamine 2000 (Invitrogen) according to the manufacturer's instructions and the recommended reagent‐to‐DNA (or siRNA) ratio. All transfection procedures were carried out strictly following the protocols provided in the instruction manual.

### 2.12 Dual‐Luciferase Reporter Assay

For the dual‐luciferase reporter assay, cells were seeded into 24‐well plates and transfected with plasmids containing the firefly luciferase reporter construct along with the Renilla luciferase internal control vector. After 24–48 h of transfection, cells were lysed using 100 µL of lysis buffer, and the supernatant was collected. Luciferase activity was measured using the Dual‐Luciferase Reporter Assay Kit (Beyotime, Shanghai, and China) according to the manufacturer's instructions. Briefly, 20 µL of cell lysate was transferred to a luminometer tube, followed by the sequential addition of 100 µL of Firefly Luciferase Assay Reagent II (LAR II) to determine firefly luciferase activity, and 100 µL of Stop & Glo Reagent to measure Renilla luciferase activity. Relative promoter activity was calculated as the ratio of firefly to Renilla luciferase luminescence.

### 2.13 Separation of Nucleus and Cytoplasm

Nucleoplasm and cytoplasm fractions were prepared using the Nucleus and Cytoplasm Separation Kit (Beyotime Biotechnology, Shanghai, China) according to the manufacturer's instructions. Briefly, 1 × 10^7^ cells were collected and washed twice with PBS. Cells were then resuspended in cell lysis buffer containing appropriate protease inhibitors and incubated on ice for 10 min. The lysate was centrifuged at 800–1000 g for 5 min to separate the cytoplasmic fraction (supernatant) from the nuclear pellet. The nuclear pellet was subsequently lysed in ice‐cold nuclear lysis buffer and incubated on ice for 10 min. Following high‐speed centrifugation at 12 000 g for 10 min, the soluble nuclear fraction was collected. Both cytoplasmic and nuclear fractions were retained for downstream analyses.

### 2.14 HPLC‐MS Detection of TSN Concentration

The reference substance of toosendanin was accurately weighed and dissolved in methanol to prepare a reference substance mother liquor containing 0.394 mg/mL toosendanin. The reference mother liquor was then accurately transferred to a 10 mL volumetric flask, diluted to the scale with methanol to prepare a reference solution containing 41.505 µg/mL toosendanin, and further diluted with methanol to prepare six different concentrations of standard solutions. The mass concentrations of toosendanin were 5188.2, 2594.1, 2075.3, 1037.6, 518.8 and 259.4 ng/mL, respectively, to assess linearity.

Chromatographic conditions: Poroshell 120 SB C18 column (2.1 mm i.d. × 100 mm, 1.8 µm). Mobile phase composition: A‐B (A is 0.1% aqueous formic acid solution, B is acetonitrile). gradient elution: 0–1 min, 10%–30% B; 1–3.5 min, 30%–40% B; 3.5–6.5 min, 40% ‐75% B; 6.5–7 min, 75%–95% B; 7–7.5 min, 95% B. column temperature: 35°C. Flow rate: 0.3 mL/min. Injection volume: 1 µL.

Mass spectrometry conditions: electrospray ion source (ESI), negative ion, turbine spray temperature 550°C, nebulizer pressure 55 psi, ion spray voltage 4500 V, heater pressure 55 psi, collision pressure medium, air curtain pressure 35 psi, opening interface heater, monitoring of reaction detection mode (MRM).

All TSN‐containing samples to be tested were dissolved in methanol by sonication, filtered through a membrane, and prepared as a clarified test solution.

### 2.15 RNA Extraction and Quantitative Reverse Transcriptase‐PCR (q‐PCR)

RNA extraction from cells: Add 1 mL trizol to the cells, mix well to disrupt the cells and cut the DNA, stand for 5 min, add 0.2 mL chloroform, shake vigorously for 30 s and stand for 10 min. The RNA is mainly in the aqueous phase, about 600 µL. The supernatant is carefully aspirated into a new 1.5 mL DEPC‐treated EP tube, 0.5 mL isopropanol is added to gently mix the liquid and allowed to stand at room temperature for 10 min. Thoroughly clean the sediment with 75% ethanol and discard the supernatant. Add an appropriate amount (20 µL) of DEPC (RNA‐free) water‐soluble RNA. Reverse transcription RNA was used to generate CDNA using a cDNA synthesis kit. Real‐time quantitative PCR was performed on Bio‐Rad C1000 using 2 × SYBR green PCR mix. All primer sequences are listed in Table S2.

### 2.16 Western Blotting

Cells were collected after drug treatment. Cells were lysed RIPA lysates (containing cocktail) on 0°C for 30–40 min. Then the supernatant was obtained by centrifugation of 13 000 rpm for 10–15 min. The supernatant (protein) content was detected by the BCA method. Proteins were separated by 6%–12% separation gel. After the end of film transfer, 5% BSA was sealed for 1 h. Use the first antibody to incubate at 4°C all night. Then incubate with the second antibody for 1 h, and wash with PBST for 3 times before color development.

### 2.17 Melanoma Tissue Collection and Immunohistochemical Staining (IHC)

Melanoma tissues were collected from the Department of Pathology of the First Hospital of Nanjing, China, as a previous sample study, and this study was approved by the Ethics Committee. (Ethical approval number: KY20250120‐KS‐06). Fixed tissue samples are paraffin‐embedded, sectioned and dewaxed for hydration, then antigenically repaired to expose antigenic sites; myeloperoxidase is removed using hydrogen peroxide; subsequently, non‐specific binding is blocked by sealing with a blocking solution for 10 min, and the primary antibody is incubated overnight at 4°C for specific recognition of the target antigen; the sample is washed three times in PBS, and the secondary antibody or a detection antibody with an enzyme/fluorescence marker is added. Finally, the target proteins are visualized by addition of the Finally, the target protein is visualized by the addition of a chromogenic substrate (DAB), and the staining results are observed using a microscope.

### 2.18 Microscale Thermophoresi (MST)

To begin, cultivate an appropriate number of 293T cells. Once the cells have grown to approximately 80% confluence, transfect them with the EGFP‐ plasmid. After 36 h, collect the cells and lyse them to extract the protein fused with enhanced green fluorescent protein (EGFP). Set aside an appropriate amount of the protein for later use. Next, dissolve TSN in a suitable buffer to ensure its purity and stability. Prepare a series of TSN solutions with concentration gradients, starting with a maximum concentration of 200 µM and diluting accordingly. Mix protein with the different concentrations of TSN solutions in microcentrifuge tubes to ensure thorough mixing. Incubate the mixtures at a suitable temperature for a specified period to allow for complete interaction between the protein and the ligand. Once prepared, load the mixtures into MST‐specific capillary tubes or assay chips. Start the MST instrument and configure the appropriate temperature, laser power, and other parameters. The MST instrument detects the thermophoretic behavior of the protein under a slight temperature gradient. If TSN interacts with protein, this interaction will alter the protein's thermophoretic behavior, which will be reflected in the measured signal. Finally, analyze the data output from the MST instrument to determine whether an interaction between TSN and protein is present. The strength of this interaction is quantified by calculating the binding constant (Kd value). A lower Kd value indicates a stronger affinity between TSN and protein.

### 2.19 Haematoxylin‐Eosin (H&E) Staining

Paraffin‐embedded tissue sections were deparaffinised and hydrated with gradient alcohol (2–5 min per step), followed by staining with hematoxylin stain for 5–10 min, then rinsed under running water for 1–2 min to remove excess dye and returned to blue under running water for 5 min after ethanol differentiation in 1% hydrochloric acid for 3–10 s; followed by staining with eosin stain for 1–3 min, and rinsed under running water again for 1 min; and, finally, dehydrated step by step (gradient alcohol 2–5 min per step), transparency (xylene 5 min per step) and sealing. The staining results were observed using a microscope.

### 2.20 Stable Cell Line Establishment

The shRNA target sequence was designed according to the BNC2 gene sequence (shBNC2 target sequence: CCGGGCAGTCCAACGTGGTGTTTGACTCGAGTCAAACCACCACGTTGGACTGCTTTTTT), followed by the design of the PCR primers (enzyme cleavage sites: AgeI and EcoRI, the specific sequences are as follows: CCGG‐GCAGTCCAACGTGGTGTTTGA‐CTCGAG‐TCAAACACCACACGTTGGACTGC‐TTTTTT). The target sequence was amplified and cloned into the pLKO.1‐mCherry‐Puro vector to generate the lentiviral shBNC2 expression plasmid. A negative control plasmid (NC‐shRNA) was also constructed to validate the specificity of shRNA‐mediated knockdown and to serve as a reference for functional analyses. All constructed plasmids were verified by sequencing. Lentiviral packaging was performed in 293T cells by co‐transfecting the shRNA plasmid together with pSPAX2 and pMD2G plasmids. Transfections were performed according to the manufacturer's protocol. 8 h after transfection, the cells were washed with 10 mL of PBS per dish, followed by replacement of the medium with fresh culture medium. Cells were then incubated at 37°C with 5% CO_2_ for 48 h. The viral supernatant was collected, centrifuged at 2000 rpm for 10 min to remove cell debris, and concentrated using a lentivirus concentration kit (Yeasen Biotech, Beijing, China). The concentrated virus was stored at ‐80°C. B16‐F10 cells were infected with the concentrated lentivirus, and puromycin was added 48 h post‐infection to select for cells stably expressing the exogenous gene.

### 2.21 Preparation of TSN Nanoliposomes

Appropriate amounts of phospholipids (e.g., lecithin, distearoyl phosphatidylcholine) and cholesterol were weighed and mixed at a defined molar ratio. The lipids and TSN compound were dissolved in an appropriate organic solvent (e.g., chloroform or a methanol‐chloroform mixture) to ensure uniform dispersion. The resulting mixture was transferred to a round‐bottom flask, and the solvent was evaporated under reduced pressure using a rotary evaporator at a temperature below 40°C, forming a homogeneous lipid film on the inner wall of the flask. A pre‐prepared hydration solution (e.g., buffer, PBS, or drug solution) was added to the lipid film, and the mixture was incubated at 37°C for 30 min with intermittent shaking or sonication to promote the formation of multilamellar liposomes (MLVs). To achieve uniform liposome size, the MLVs were further processed into monodisperse large unilamellar vesicles (LUVs) using probe or bath sonication, or by repeated extrusion through polycarbonate membranes of defined pore size. The resulting liposomes were purified by ultracentrifugation to remove unencapsulated free compounds. Liposome morphology, particle size distribution, and dynamic behavior were characterized using NanoSight Pro and dynamic light scattering (DLS, Malvern Panalytical, UK), while the encapsulation efficiency and drug loading were determined by HPLC‐MS. The final liposome suspensions were stored at 4°C protected from light.

### 2.22 Establishment and Treatment of Mouse Tumor Model

Establishment of mouse lung metastasis model: B16‐F10 melanoma cells were cultured under standard conditions and harvested at the logarithmic growth phase. After cell counting, the suspension was adjusted to a final concentration of 5 × 10^3^ or 1 × 10^4^ cells/µL in PBS. A total of 100 µL of the cell suspension (5 × 10^5^ or 1 × 10^6^ cells) was injected slowly into the lateral tail vein of C57BL/6 mice using a 1 mL insulin syringe. After injection, the site was gently pressed with a sterile cotton ball for several seconds to prevent bleeding and ensure proper closure. Mouse melanoma lung normal metastasis model: 5 × 10^5^ B16‐F10 cells. Mouse melanoma lung high metastasis model: 1 × 10^6^ B16‐F10 cells.

Mouse subcutaneous in situ tumor model: B16‐F10 cells were cultured and collected in good condition at the logarithmic phase. After counting, the cells were resuspended in PBS at a concentration of 5 × 10^3^ cells/µL. A total of 100 µL of the suspension (5 × 10^5^ cells) was injected subcutaneously into the right flank or dorsal region of the mice. The injection was performed swiftly to prevent leakage, and gentle pressure with a sterile cotton ball was applied for several seconds to ensure proper wound sealing.

All animal experiments were approved by the Science and Technology Ethics Committee of Nanjing University (Approval No: IACUC‐2503002).

The TSN administration method in all models above is intraperitoneal injection. The TSN group (0.1, 0.5, 1.0 mg/kg) solution is prepared as follows: 10% DMSO + 40% PEG300 + 5% Tween‐80 + 45% Saline. The control group (0.0 mg/kg) has the same solvent composition as above except for the absence of TSN.

### 2.23 Comparative Pharmacokinetic Study of Free TSN and TSN Nanoliposomes

Male C57BL/6 mice were intravenously administered free TSN or TSN‐ nanoliposomes (TSN‐nano) at a dose of 2 mg/kg via the tail vein. Blood samples (300–500 µL) were collected into heparinized tubes at predetermined time points (5 min, 15 min, 30 min, 1 h, 2 h, 4 h, 8 h, 12 h, and 24 h), and plasma was separated by centrifugation at 4000 × g for 10 min at 4°C. Plasma proteins were precipitated with methanol containing acetaminophen as the internal standard. The supernatant was evaporated to dryness under a nitrogen stream, reconstituted with methanol, and filtered through a 0.22 µm membrane filter prior to injection. TSN concentrations were determined using the LC‐MS/MS method described in Section 2.14. Pharmacokinetic parameters were calculated by non‐compartmental analysis: AUC was computed using the linear trapezoidal rule; λz was estimated by log‐linear regression of the terminal phase; t_1_/_2_ = ln (2)/λz; MRT was calculated as AUMC/AUC; and the relative bioavailability was determined as (AUC_0__−__∞_, TSN‐nano / AUC_0__−__∞_, TSN) × 100%. Data were expressed as mean ± SEM.

### 2.24 Preparation of DiR‐Labeled Nanoliposomes and In Vivo Near‐Infrared Fluorescence Imaging

DiR was dissolved in anhydrous DMSO or ethanol at 1–2 mg/mL and added dropwise to pre‐formed nanoliposomes at 1 mol% of total lipid, followed by incubation in the dark at room temperature for 30 min (or 37°C for 15–30 min) to allow DiR insertion into the lipid bilayer. Unbound dye was removed by dialysis (dialysis bag, Beijing Solarbio Science & Technology Co., Ltd., Beijing, China) against ≥100 volumes of PBS (pH 7.4) at 4°C under gentle stirring for 12–24 h with buffer replacement every 2–4 h, and removal was confirmed by stable fluorescence in the outer solution. The liposome suspension was filter‐sterilized (0.22 µm). For in vivo imaging, female C57BL/6 mice (6–8 weeks old) were inoculated subcutaneously with B16‐F10 melanoma cells in the upper right hind flank, and tumors were grown to ∼100–200 mm^3^. Mice were randomly divided into the TSN‐nanoliposome‐DiR group and the nanoliposome‐DiR control group (n ≥ 5 per group), and received equivalent doses via tail vein injection. At 2, 6, and 12 h post‐injection, mice were anesthetized (isoflurane) and imaged with an IVIS system (excitation 745–750 nm, emission 780–800 nm). At the terminal time point, heart, liver, spleen, lungs, and kidneys were excised and imaged ex vivo.

### 2.25 Statistical Analysis

Statistical analyses were performed using SPSS software (version 16.0; SPSS, Inc., Chicago, IL, USA) and Prism software (version 8.0; GraphPad, Inc., San Diego, CA, USA). All data were presented as mean ± SEM and experiments were repeated at least three independent times. Comparisons between two groups were performed using two‐tailed Student's t‐test (data with Gaussian distribution). Comparisons between multiple groups were made by One‐way ANOVA with Tukey's post‐hoc test was used to compare each column mean with every other column mean. Statistical significance was defined as p < 0.05 (^*/#^ *p* < 0.05; ^**/##^ *p* < 0.01; ^***/###^ *P*< 0.001, ^****/####^ *P*< 0.0001).

## 3 Results

### 3.1 TSN Suppresses Melanoma Cell Growth and Promotes Apoptosis

**FIGURE 1:**
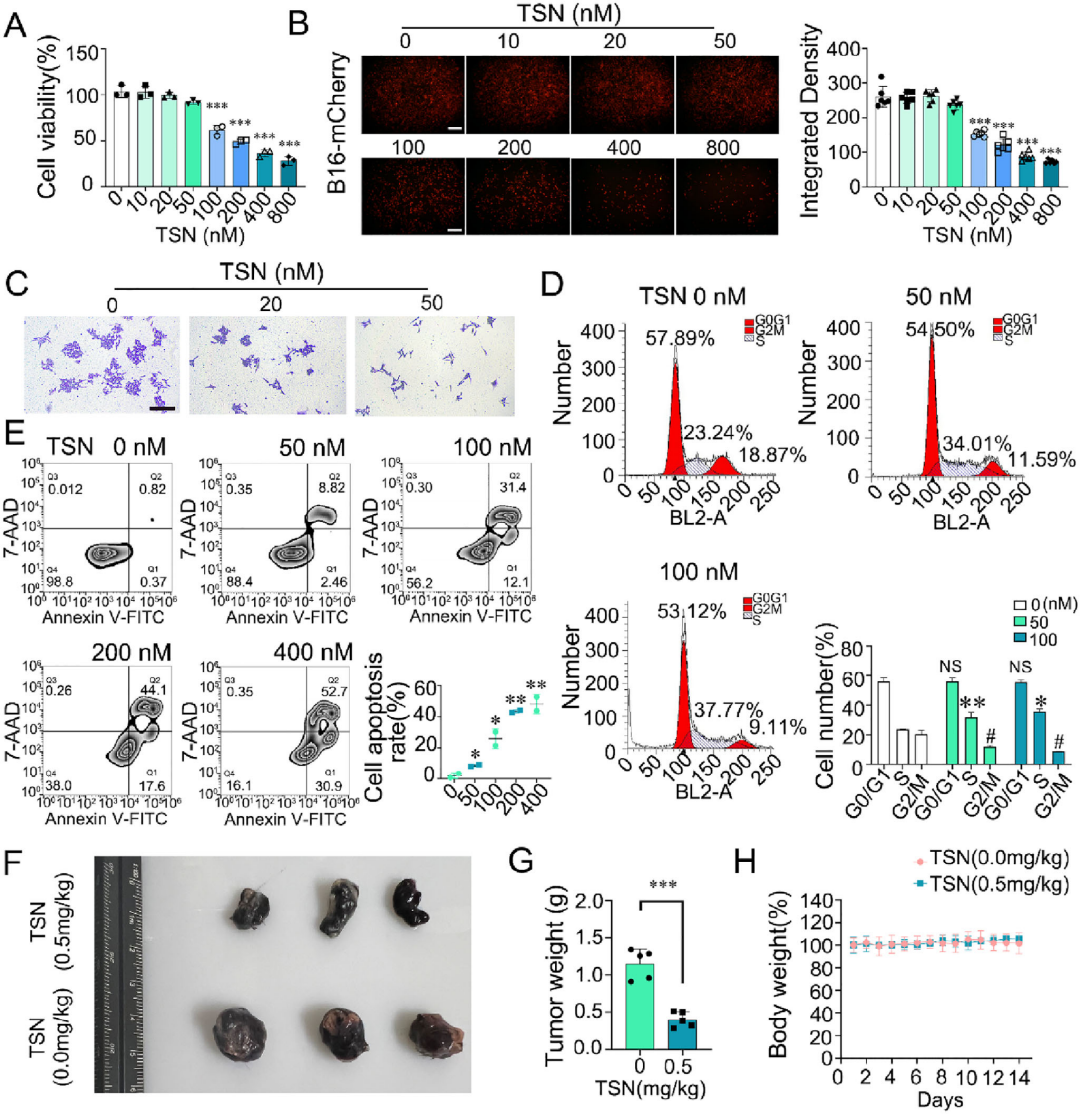
TSN inhibits melanoma cell proliferation. (A) MTT assay was used to assess cell viability of B16‐F10 cells after treatment with different concentrations of TSN (0, 10, 20, 50, 100, 200, 400, and 800 nM), n = 3. (B) Fluorescence analysis of B16‐F10‐mCherry cells treated with TSN (0, 10, 20, 50, 100, 200, 400, and 800 nM) to evaluate cell proliferation, n = 6. Scale bar: 200 µm. (C) Colony formation assay showing the clonogenic ability of B16‐F10 cells after treatment with TSN (0, 20, and 50 nM). (D) Cell cycle analysis of B16‐F10 cells treated with TSN (0, 50, and 100 nM) using a cell cycle detection kit, n = 2. (E) Apoptosis analysis of B16‐F10 cells treated with different concentrations of TSN (0, 50, 100, 200, and 400 nM) analyzed by flow cytometry, n = 2. (F) Representative images of resected subcutaneous tumors from C57BL/6 mice treated with TSN (0.0 mg/kg and 0.5 mg/kg), n = 5. (G) Tumor weights from subcutaneous melanoma‐bearing mice after intraperitoneal injection of TSN (0.0 mg/kg and 0.5 mg/kg), n = 5. (H) Body weights of mice bearing subcutaneous melanoma tumors following intraperitoneal injection of TSN (0.0 mg/kg and 0.5 mg/kg), n = 5. Statistical significance was defined as ^*^ *p* < 0.05, ^**^ *p* < 0.01 and ^***^ *p* < 0.001. ^#^ *P*< 0.05.

To evaluate the anti‐tumor efficacy of TSN, a series of in vitro and in vivo experiments were conducted using B16‐F10 melanoma cells. TSN treatment significantly decreased cell viability in a dose‐dependent manner, as shown by MTT assays (Figure 1A). Consistently, fluorescence imaging of B16‐F10‐mCherry cells revealed a pronounced reduction in cell proliferation following TSN exposure (Figure 1B). TSN markedly suppressed the colony‐forming ability of B16‐F10 cells (Figure 1C). Flow cytometric analysis further demonstrated that TSN induced S‐phase arrest, as evidenced by an increased S‐phase fraction and a concomitant reduction in the G2/M population (Figure 1D). In parallel, TSN induced apoptosis in a dose‐dependent manner (Figure 1E).

To validate these findings in vivo, we established a subcutaneous B16‐F10 melanoma model in C57BL/6 mice. TSN markedly inhibited tumor growth (Figure 1F and Figure S1A) and significantly reduced tumor weight (Figure 1G). Importantly, no significant change in body weight was observed in TSN‐treated mice, suggesting that TSN treatment did not cause overt systemic toxicity at the administered dose (Figure 1H). Collectively, these findings demonstrate that TSN effectively inhibits melanoma cell proliferation and tumor growth both in vitro and in vivo.

### 3.2 TSN Inhibits the Metastatic Potential of Melanoma

**FIGURE 2:**
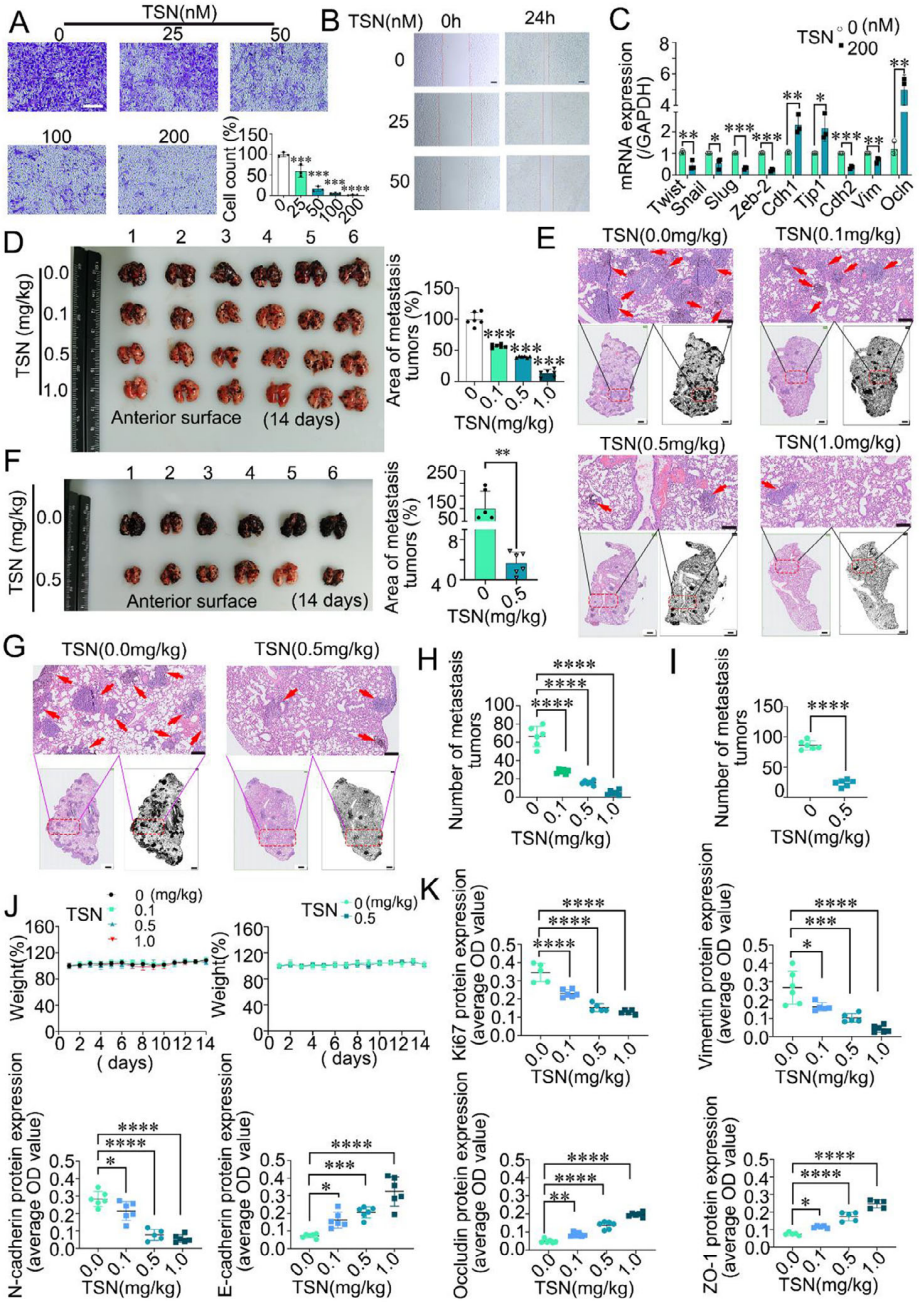
TSN significantly inhibits B16‐F10 metastasis. (A) Transwell representative images and statistical results show the migratory capacity of B16‐F10 cells treated with TSN (0, 25, 50, 100, and 200 nM). Scale bar: 200 µm. (B) Scratch wound assay was used to evaluate the migratory ability of B16‐F10 cells treated with TSN (0, 25, and 50 nM). Scale bar: 100 µm. (C) qPCR analysis of *Twist*, *Snail*, *Slug*, *Cdh1*, *Tjp1*, *Cdh2*, *Vim* and *Ocln* mRNA expression in B16‐F10 cells treated with 200 nM TSN, n = 3. (D) Representative images of lungs from the melanoma lung metastasis mouse model treated with TSN (0.0, 0.1, 0.5, 1 mg/kg) and quantification of melanoma lesion area in the lungs of the melanoma lung metastasis model after TSN treatment, n = 6. (E) H&E staining of lung tissues in the mouse melanoma lung metastasis model after intraperitoneal injection of TSN. Scale bar: 1000 and 200 µm. (F) Representative lung images from the highly metastatic melanoma model treated with TSN (0.5 mg/kg) and quantification of melanoma lesion area in the lungs of the high‐metastasis melanoma model after TSN treatment, n = 6. (G) H&E staining of lung tissues in the mouse melanoma lung high metastasis model after intraperitoneal injection of TSN. Scale bar: 1000 and 200 µm. (H—I) Quantification of pulmonary metastatic nodules in both melanoma metastasis models, n = 6. (J) Body weights of the mouse melanoma lung metastasis model and high metastasis model after intraperitoneal injection of TSN, n = 6. (K) Representative IHC staining statistical results of Ki‐67, E‐cadherin, N‐cadherin, Vimentin, Occludin, and ZO‐1 expression after TSN treatment in a mouse melanoma lung metastasis model, n ≥ 5. Statistical significance was defined as ^*^ *p* < 0.05, ^**^ *p* < 0.01, ^***^ *p* < 0.001, and ^****^ *p* < 0.0001.

We next investigated whether TSN suppresses the metastatic potential of melanoma cells. in vitro, transwell and scratch assays showed TSN markedly suppressed B16‐F10 cell migration and invasion (Figure 2A,B and Figure S1B). Furthermore, TSN downregulated the mRNA expression of key metastasis‐associated transcription factors (*Twist*, *Snail*, *Slug, Zeb‐2*, *Cdh2*, *Vim*) while upregulating cell‐cell junction proteins (*Cdh1*, *Tjp1*, *Ocln*) mRNA expression (Figure 2C). To evaluate the anti‐metastatic activity of TSN in vivo, a B16‐F10 melanoma lung metastasis model was established by tail vein injection [27]. TSN treatment significantly reduced the area of metastatic nodules on both the anterior and posterior lung surfaces in a dose‐dependent manner (Figure 2D and Figure S1C,D). Consistently, H&E staining revealed markedly fewer and smaller pulmonary metastatic lesions in TSN‐treated mice compared with controls (Figure 2E). The potent anti‐metastatic effect of TSN has been further validated in the B16‐F10 highly metastatic melanoma model, where TSN again significantly inhibited melanoma metastasis (Figure 2F and Figure S1E). The results of H&E staining confirmed that there were fewer and smaller pulmonary metastatic lesions after TSN treatment (Figure 2G). Quantification of H&E‐stained metastatic foci in both models demonstrated the robust anti‐metastatic efficacy of TSN (Figure 2H,I). Notably, TSN‐treated mice showed no significant loss of body weight or evidence of hepatic toxicity (Figure 2J and Figure S1F–H). In addition to pulmonary metastases, renal metastases were also observed in the B16‐F10 highly metastatic melanoma model, which were significantly abrogated by TSN treatment (Figure S1I). Immunohistochemical analysis further confirmed that TSN treatment markedly reduced the expression of Ki‐67, N‐cadherin, and Vimentin, while enhancing the expression of E‐cadherin, Occludin, and ZO‐1 in lung metastatic lesions (Figure 2K and Figure S2A). Furthermore, immunostaining with the melanoma‐specific marker S‐100 further confirmed that the metastatic foci in the lung tissue originated from melanoma (Figure S2B). Taken together, these findings demonstrate that TSN exhibits potent anti‐metastatic activity against melanoma in vitro and in vivo.

### 3.3 TSN Targets BNC2 to Suppress *PIK3CA* Transcription in Melanoma

We previously reported that TSN is a potent PI3K inhibitor that can suppress both the transcription and protein expression levels of *PIK3CA*(p110α) in breast cancer cells [21].

**FIGURE 3:**
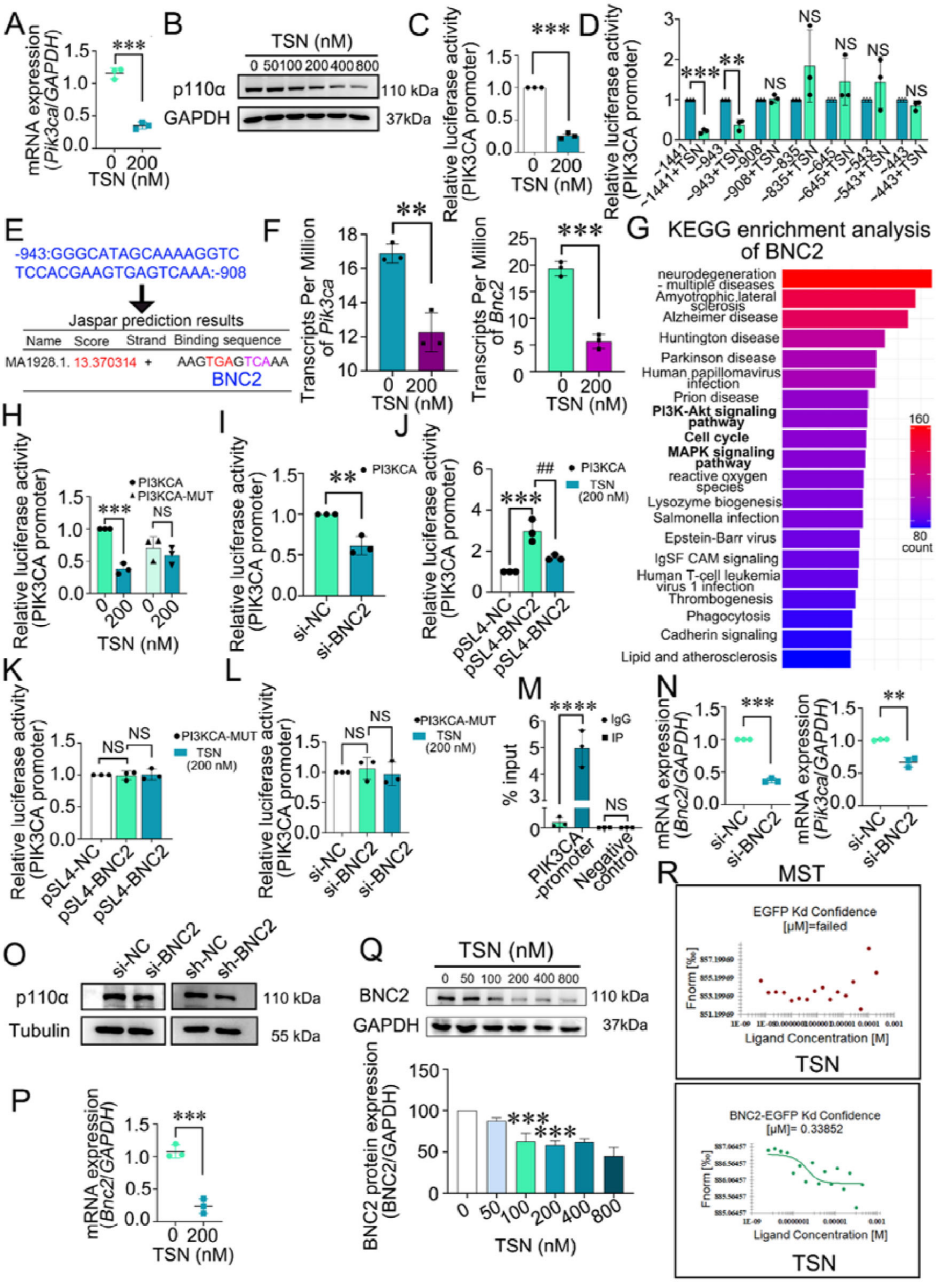
TSN inhibits the BNC2*‐PIK3CA* pathway. (A) qPCR analysis of *Pik3ca* mRNA expression in B16‐F10 cells treated with 200 nM TSN. n = 3. (B) Western blot analysis of p110α protein expression in B16‐F10 cells treated with different concentrations of TSN. (C) 293T cells were transfected with 1 µg of *PIK3CA* promoter plasmids, 0.1 µg of pRL plasmid, and co‐treated with 200 nM TSN for 36 h. Luciferase activity was measured following standard protocols using a pGL3‐basic promoter reporter plasmid as the control, n = 3. (D) Construction of *PIK3CA* promoter deletion plasmids. 293T cells were transfected with 1 µg of *PIK3CA* promoter plasmids containing different deletions, along with 0.1 µg of the pRL plasmid, and co‐treated with 200 nM TSN for 36 h. Luciferase activity was measured following standard protocols, using the pGL3‐basic promoter reporter plasmid as a control, n = 3. (E) Prediction of highly bound transcription factors on the *PIK3CA* promoter ‐943 to ‐908 sequence using the JASPAR transcription factor database, and BNC2 was the highest‐scoring transcription factor. (F) RNA ‐seq analysis of *PIK3CA* and *Bnc2* mRNA expression in B16‐F10 cells treated with 200 nM TSN. n = 3. (G) KEGG pathway enrichment analysis of *Bnc2*. (H) Construction of a *PIK3CA* promoter TGA‐TCA mutation reporter plasmid. 293T cells were transfected with 1 µg of *PIK3CA* promoter plasmids or *PIK3CA* mutation promoter, 0.1 µg of pRL plasmid, and co‐treated with 200 nM TSN for 36 h. Luciferase activity was measured following standard protocols using a pGL3‐basic promoter reporter plasmid as the control, n = 3. (I—L) 293T cells were transfected with 1 µg of *PIK3CA* promoter plasmids or *PIK3CA* mutation promoter (*PIK3CA‐*MUT), 0.1 µg of pRL plasmid, and then transfected with 1 µg pSL4‐BNC2, si‐BNC2, respectively, and co‐treated with 200 nM TSN for 36 h. Luciferase activity was measured following standard protocols using a pGL3‐basic promoter reporter plasmid as the control, n = 3. (M) ChIP‐qPCR analysis of BNC2 enrichment at the *PIK3CA* promoter region. Data are presented as percentage of input (% Input) relative to input DNA. n = 3. (N) qPCR analysis of *Bnc2* and *PIK3CA* mRNA expression in B16‐F10 cells transfected with si‐BNC2, n = 3. (O) Western blot analysis of p110α protein expression following BNC2 knockdown. (P) qPCR analysis of *Bnc2* and *PIK3CA* mRNA expression in B16‐F10 cells treated with varying concentrations of TSN, n = 3. (Q) Western blot analysis of BNC2 protein expression in cells treated with different concentrations of TSN, n = 3. (R) Representative images of microscale thermophoresis (MST) in the EGFP and BNC2‐EGFP groups; TSN binds to BNC2 with an affinity Kd value of 0.33852 µM (mean ± 95% CI, n = 3). Statistical significance was defined as ^**^ *p* < 0.01, ^***^ *p* < 0.001, and NS> 0.05. ^##^ *p* < 0.01.

Further, in contrast to traditional PI3K inhibitors that suppress the activity of the PI3K enzyme, TSN specifically inhibits the expression of the *PIK3CA* gene in cancerous cells while leaving normal cells unaffected. To determine whether the anti‐tumor effect of TSN on melanoma involves PI3K pathway suppression, we examined the expression of *PIK3CA*. TSN treatment led to a marked reduction in the mRNA levels of *Pik3ca*, the gene encoding the p110α protein (Figure 3A). TSN treatment also reduced p110α protein expression in a dose‐ dependent manner (Figure 3B). To delineate the underlying mechanisms, we constructed a dual‐luciferase reporter (pGL3‐*PIK3CA*) driven by the *PIK3CA* promoter and found that TSN markedly suppressed promoter activity (Figure 3C), supporting the notion that TSN suppresses *PIK3CA* transcription. By generating a series of truncated pGL3‐*PIK3CA* reporters that sequentially removed putative transcription factor binding sites, we pinpointed the TSN‐responsive region cis‐elements to the region between −943 and −908 in the promoter (Figure 3D). Transcription factor prediction using the JASPAR database identified basonuclin 2 (BNC2) as the highest‐scoring candidate binding to this region, which contains conserved TGA and TCA motifs (Figure 3E). RNA‐seq analysis revealed that TSN treatment significantly downregulated *PIK3CA* mRNA expression (Figure 3F). Notably, *BNC2* transcript levels were also markedly reduced (Figure 3F). KEGG pathway enrichment analysis further demonstrated that *BNC2* is closely associated with neuro‐related disease pathways, the PI3K‐AKT signaling pathway, the cell cycle pathway, and the MAPK signaling pathway (Figure 3G). These results further establish a close relationship among TSN, *BNC2*, and *PIK3CA*.

Site‐directed mutagenesis of the BNC2‐binding motif abolished the inhibitory effect of TSN on promoter activity (Figure 3H), demonstrating that BNC2 is essential for TSN‐mediated transcriptional repression of *PIK3CA*. Crucially, this finding also confirms that BNC2 is a key transcriptional activator of *PIK3CA*. Consistent with this finding, BNC2 knockdown markedly decreased pGL3‐*PIK3CA* promoter activity (Figure 3I), whereas BNC2 overexpression produced an increase in promoter activity (Figure 3J). TSN effectively inhibited the BNC2 overexpression—mediated induction of PIK3CA promoter activity (Figure 3J). However, when the BNC2 binding site within the promoter was mutated, BNC2 lost its regulatory effect on promoter activity, regardless of whether it was knocked down or overexpressed (Figure 3K,L). To further determine whether BNC2 directly binds to the *PIK3CA* promoter, we performed ChIP‐qPCR in B16 cells expressing BNC2‐FLAG. Anti‐FLAG immunoprecipitation detected significant enrichment at the *PIK3CA* promoter region, whereas no enrichment was observed at the negative control region, indicating that BNC2 specifically binds to the *PIK3CA* promoter (Figure 3M).

In B16‐F10 cells, BNC2 knockdown markedly reduced the mRNA and protein levels of *PIK3CA* (Figure 3N,O). Intriguingly, TSN treatment also significantly decreased *BNC2* mRNA and protein expression (Figure 3P,Q), consistent with the decreased *BNC2* expression observed in sequencing results. Given that BNC2 is a transcription factor capable of regulating its own transcription [16], we hypothesized that TSN may directly target BNC2 protein and promote its degradation. This hypothesis was validated by microscale thermophoresis (MST) analysis, which demonstrated that TSN can directly bind to BNC2 (Figure 3R) with a binding Kd value of 0.33852 µM. Together, these data indicate that BNC2 is a critical transcriptional activator of PIK3CA. TSN may suppress PI3K signaling by directly binding to and inhibiting BNC2.

### 3.4 BNC2 is Highly Expressed in Skin Cutaneous Melanoma and Negatively Correlated With Patient Survival

**FIGURE 4:**
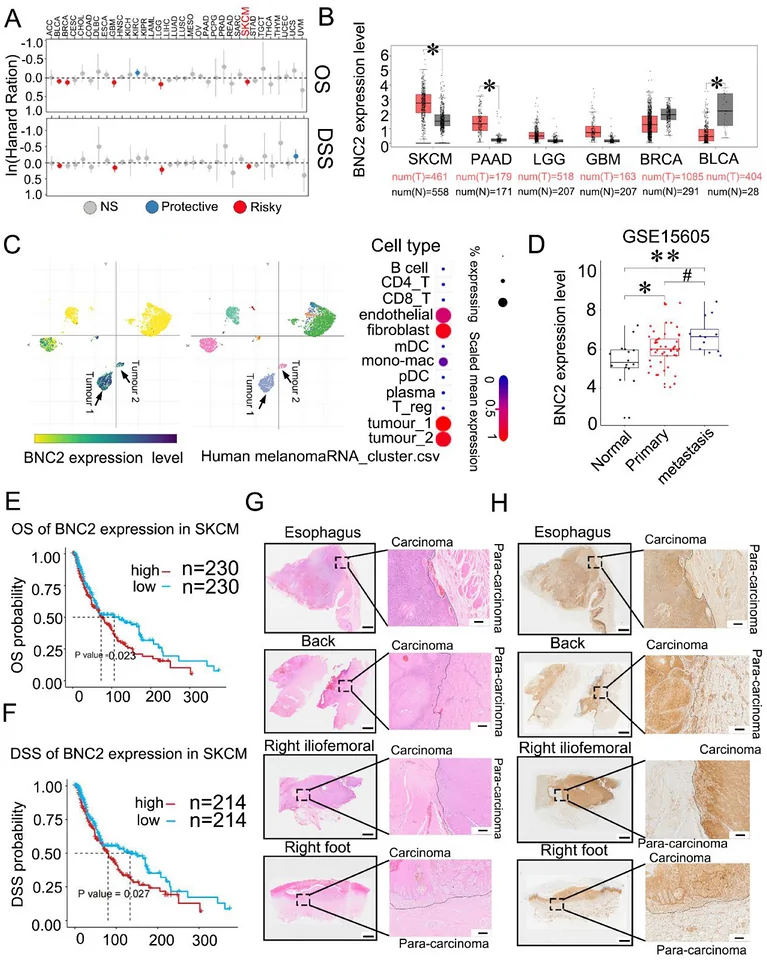
BNC2 is highly expressed in SKCM patients and predicts poor prognosis. (A) Analysis of the impact of BNC2 on survival in SKCM patients (TCGA database) by the proportional hazards model (Cox model). (B) The mRNA expression level of BNC2 among SKCM, PAAD, LGG, GBM, BRCA, and BLCA was analyzed using the GEPIA2 database. (C) Single‐cell data from melanoma tissues were used to analyze BNC2 expression in different cells using the Single Cell PORTAL database. (D) mRNA expression levels of BNC2 in normal tissue, primary SKCM, and metastatic SKCM by GEO (GSE15605) database. (E—F) Overall survival (OS) and disease‐specific survival (DSS) analyses of SKCM patients were performed using the OncoLnc database. (G—H) Representative H&E staining and BNC2 immunohistochemical staining of tumor tissue sections from SKCM patients in the clinic. Scale bar: 2 mm and 200 µm. Statistical significance was defined as ^*^ *p* < 0.05, ^**^ *p* < 0.01 and ^***^ *p* < 0.001. ^#^ *p* < 0.05.

BNC2 is a specific zinc finger protein that plays a crucial role in tumor biology [28]. However, its expression pattern and regulatory function in melanoma remain poorly defined. To clarify its clinical significance, we performed a Cox proportional hazards regression analysis to evaluate the association between BNC2 expression and patient survival across multiple cancer types using data from The Cancer Genome Atlas (TCGA) [29]. The Cox model identified BNC2 as a high‐risk factor associated with poorer overall survival (OS) and disease‐specific survival (DSS) in several cancers, including skin cutaneous melanoma (SKCM), lower‐grade glioma (LGG), glioblastoma multiforme (GBM), breast carcinoma (BRCA), and bladder carcinoma (BLCA) (Figure 4A). Among these, *BNC2* mRNA expression was markedly elevated in SKCM and pancreatic adenocarcinoma (PAAD), with the highest expression observed in SKCM (Figure 4B). Single‐cell transcriptomic profiling of normal skin and SKCM tissues further showed that BNC2 expression was predominantly localized in melanocytes and melanoma cells, underscoring its potential role in melanoma development and progression (Figure 4C and Figure S3A). Interestingly, as shown in the GSE15605 dataset [30], *BNC2* expression was substantially higher in metastatic SKCM compared to primary SKCM (Figure 4D), suggesting that BNC2 may facilitate melanoma progression and metastasis. Survival analysis revealed that higher BNC2 expression was significantly associated with poorer OS and DSS in SKCM patients (Figure 4E,F), whereas no significant prognostic association was observed in other tumors (Figure S3B—F). To further validate these findings, we examined clinically resected melanoma specimens from multiple patients. Both primary and metastatic melanoma tissues exhibited significantly higher BNC2 expression compared with adjacent non‐tumorous tissues, as demonstrated by immunohistochemistry, with representative H&E staining shown for histopathological context (Figure 4G,H and Figure S3G,H).

Pathway enrichment analysis of TCGA melanoma datasets revealed that the PI3K signaling pathway ranked among the top ten pathways most strongly associated with *BNC2* expression (Figure S4A). *PIK3CA* showed a positive correlation with BNC2 expression (Figure S4B). In addition, consistent with *BNC2* expression, *PIK3CA* expression was higher in metastatic melanoma than in primary tumors [30] (Figure S4C). Single‐cell RNA sequencing of melanoma samples further identified a cell cluster characterized by high co‐expression of *BNC2* and *PIK3CA* (Figure S4D). High p110α expression was also observed in melanoma tissue sections with high BNC2 expression (Figure S4E). Taken together, these results demonstrate that BNC2 is highly and specifically overexpressed in melanoma, correlates with disease progression and poor prognosis, and is closely associated with activation of the PI3K signaling pathway.

### 3.5 BNC2 Expression is Strongly Involved in the Proliferation and Metastasis of Melanoma Cells

**FIGURE 5:**
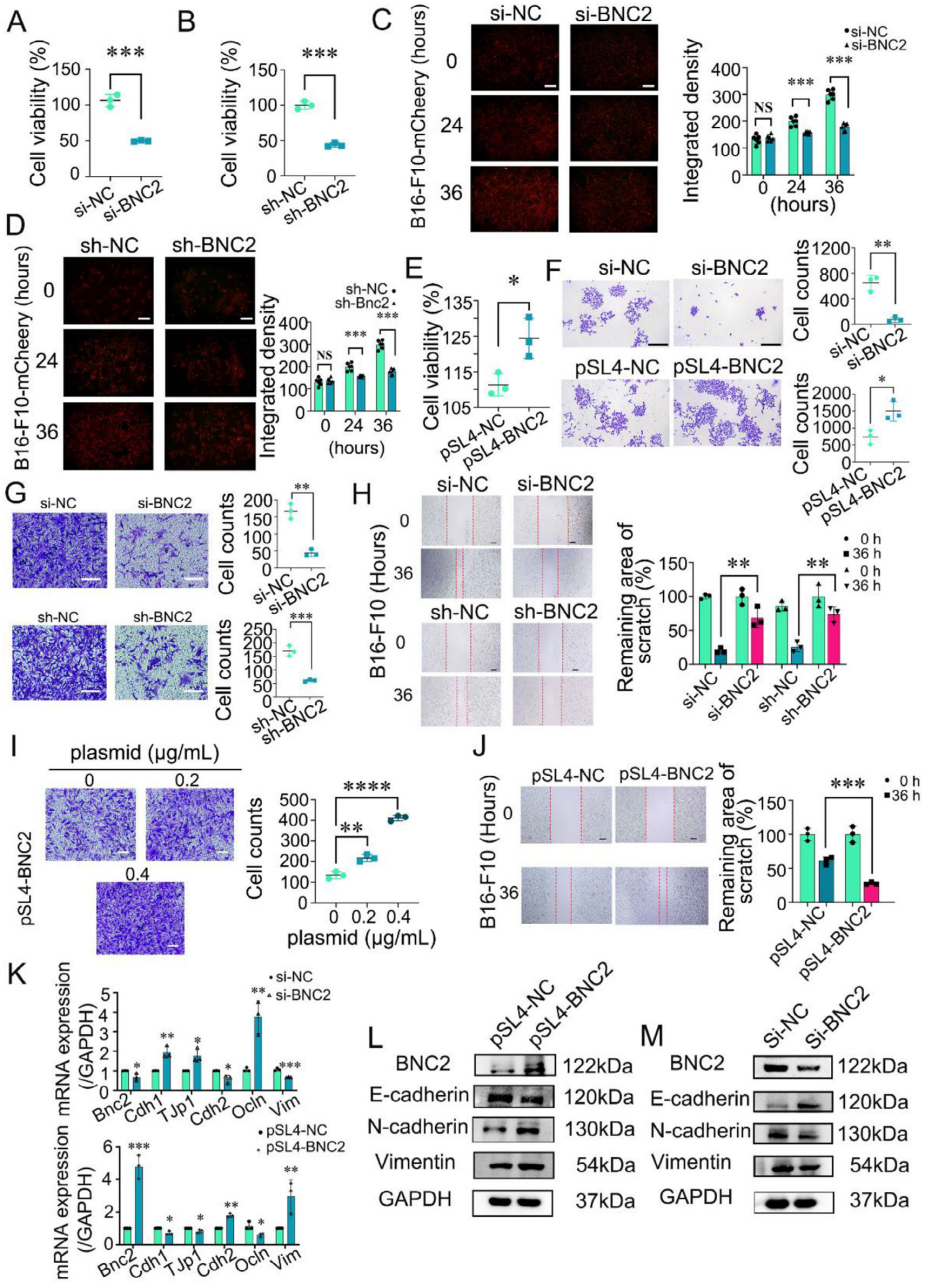
BNC2 promotes melanoma proliferation and metastasis. (A and B) MTT assay was used to assess cell viability of B16‐F10 cells after transfection of si‐BNC2 and sh‐BNC2, n = 3. (C—D) Fluorescence analysis showing cell proliferation of B16‐F10‐mCherry cells after transfection of si‐BNC2 and sh‐BNC2, n = 6. Scale bar:200 µm. (E) MTT assay detected cell proliferation ability of B16‐F10 cells after transfection of pSL4‐BNC2, n = 3. (F) The colony formation assay was utilized to assess the capacity of B16‐F10 cells to form colonies following the transfection of si‐BNC2 and PSL4‐BNC2. Scale bar:200 µm. n = 3. (G) Transwell migration assay assessing the migratory capacity of B16‐F10 cells after BNC2 knockdown (si‐BNC2, sh‐BNC2) or overexpression (pSL4‐BNC2). Scale bar: 200 µm. n = 3. (H) Scratch wound‐healing assay showing migration of B16‐F10 cells following BNC2 knockdown. Scale bar:100 µm. n = 3. (I—J) Transwell and scratch assays showing enhanced migration of B16‐F10 cells following BNC2 overexpression. Scale bar: 200 µm. n = 3. (K) qPCR to detect the mRNA expression of *BNC2*, *Cdh1*, *Tjp1*, *Vim*, *Cdh2* and *Ocln* after transfection with si‐BNC2 and pSL4‐BNC2, n = 3. (L—M) Western blot analysis of protein expression of BNC2, E‐cadherin, N‐cadherin and Vimentin after transfection with si‐BNC2 or pSL4‐BNC2.The differences were considered statistically significant with ^*^ *p* < 0.05, ^**^ *p* < 0.01, ^***^ *p* < 0.001 and ^****^ *p* < 0.0001.

To investigate the role of BNC2 in melanoma progression and metastasis, we manipulated BNC2 expression in B16‐F10 cells using small interfering RNA (si‐BNC2), short‐hairpin plasmids (sh‐BNC2), and overexpression plasmids (pSL4‐BNC2) (Figure S5A–C). Knockdown of BNC2 significantly inhibited cell viability compared to the control group, as demonstrated by MTT assays (Figure 5A,B). In B16‐F10‐mCherry fluorescent cells, we further confirmed that BNC2 knockdown substantially suppressed cell proliferation (Figure 5C,D). Conversely, BNC2 overexpression significantly promoted cell proliferation (Figure 5E). Colony formation assays showed that BNC2 knockdown substantially inhibited clonogenic capacity, while overexpression enhanced colony formation (Figure 5F). Transwell and scratch assays showed that BNC2 knockdown significantly inhibited B16‐F10 cell migration (Figure 5G,H), while forced overexpression of BNC2 markedly enhanced cell migration (Figure 5I,J). Like TSN treatment, BNC2 knockdown resulted in a significant upregulation of epithelial markers, including *Cdh1*, *Tjp1*, and *Ocln* mRNA, accompanied by a corresponding downregulation of mesenchymal markers, such as *Cdh2* and *Vim* mRNA (Figure 5K). In contrast, overexpression of BNC2 produced the opposite effects (Figure 5K). Western blot analysis confirmed these results (Figure 5L,M). Collectively, these results demonstrate that BNC2 plays a critical role in promoting melanoma cell proliferation, migratory capacity, and metastatic potential.

### 3.6 Knockdown of BNC2 Significantly Suppresses Melanoma Lung Metastasis in vivo

**FIGURE 6:**
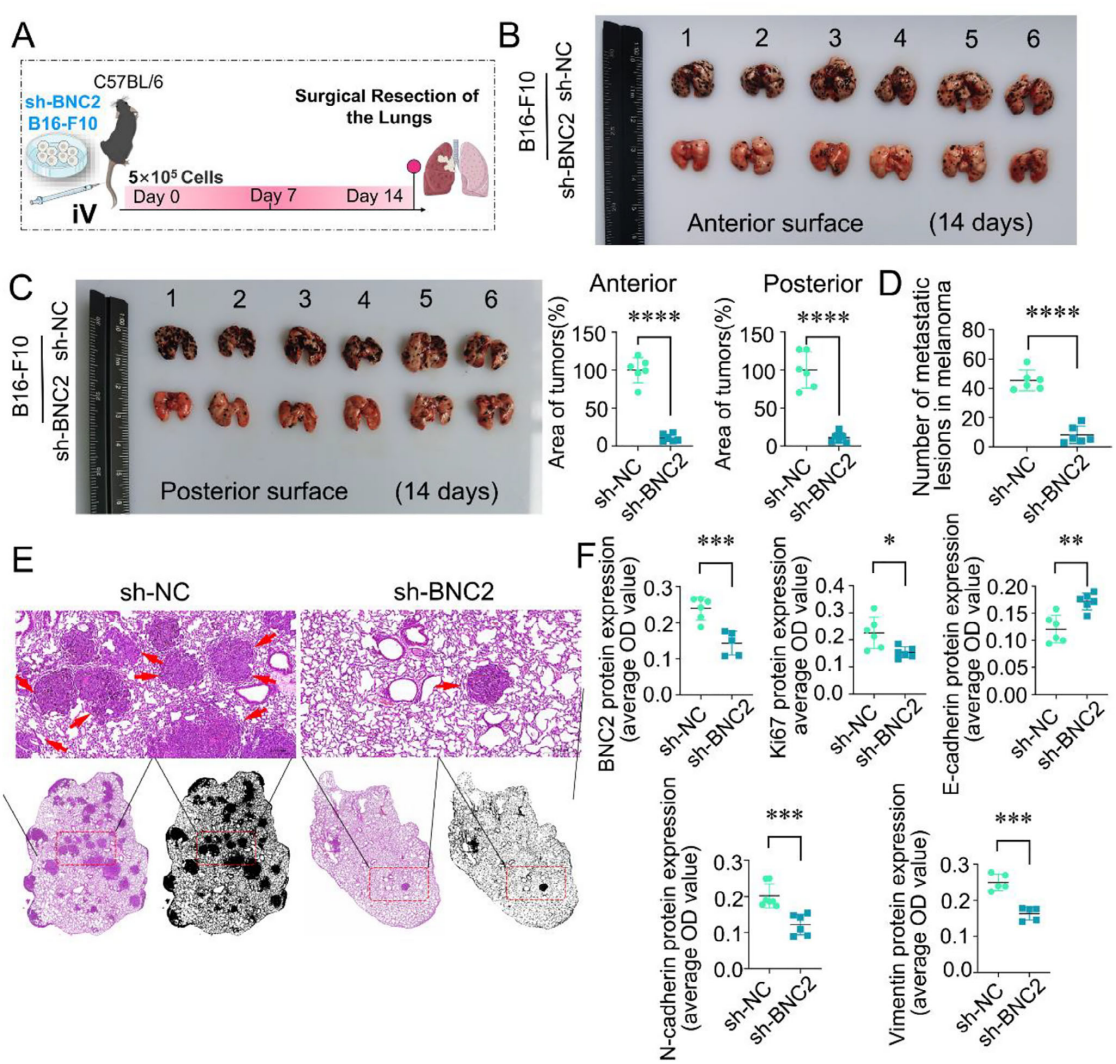
Lung metastatic capacity is significantly reduced after BNC2 knockdown in B16‐F10 cells. (A) Schematic diagram of the mouse melanoma lung metastasis model. Mice were injected via the tail vein with 5 × 10^5^ stable BNC2‐knockdown or control B16‐F10 cells. Lungs were harvested 14 days later to assess melanoma metastasis. (B—C) Representative images of lungs and quantification of metastatic lesion area in mice from the sh‐NC and sh‐BNC2 groups in the melanoma lung metastasis model, n = 6. (D—E) Quantification of pulmonary metastatic nodules and representative H&E‐stained lung sections from sh‐NC and sh‐BNC2 groups. Scale bar: 1000 and 200 µm. (F) Quantification of immunohistochemical staining for BNC2, Ki‐67, E‐cadherin, N‐cadherin, and Vimentin in lung metastatic lesions from sh‐NC and sh‐BNC2 mice, n = 6. Statistical significance was defined as ^*^ *p* < 0.05, ^**^ *p* < 0.01, ^***^ *p* < 0.001, and ^****^ *p* < 0.0001.

Given that BNC2 plays a critical role in promoting B16‐F10 cell proliferation and metastasis, we continued to examine its effect in vivo. Previous studies have indicated that complete BNC2 knockout in mice is embryonic lethal [31]; we therefore constructed a B16‐F10 cell line with reduced BNC2 expression by shRNA‐mediated gene silencing for further experimentation. Western blot analysis revealed that shRNA delivery resulted in a significant decline in BNC2 expression in B16‐F10 cells (Figure S5D,E). Subsequently, a mouse model of melanoma metastasis to the lung was developed using a tail vein injection (Figure 6A). The results demonstrated that the metastatic potential of B16‐F10 cells to the lung was significantly reduced following BNC2 knockdown (Figure 6B,C and Figure S5F). Additionally, H&E staining further confirmed further corroborated the substantial decrease in metastatic nodules in the lungs of mice with BNC2 knockdown (Figure 6D,E). Immunohistochemical analysis demonstrated that proliferation and metastasis markers, including Ki‐67, Vimentin, and N‐cadherin, were significantly downregulated, whereas E‐cadherin expression was markedly upregulated (Figure 6F and Figure S5G). No liver or kidney metastases were detected under these experimental conditions (Figure S5H,I). Collectively, these results indicate that BNC2 knockdown markedly suppresses the metastatic capacity of melanoma cells in vivo.

### 3.7 TSN Targets BNC2 and Promotes its Degradation in both the Cytoplasm and Nucleus

**FIGURE 7:**
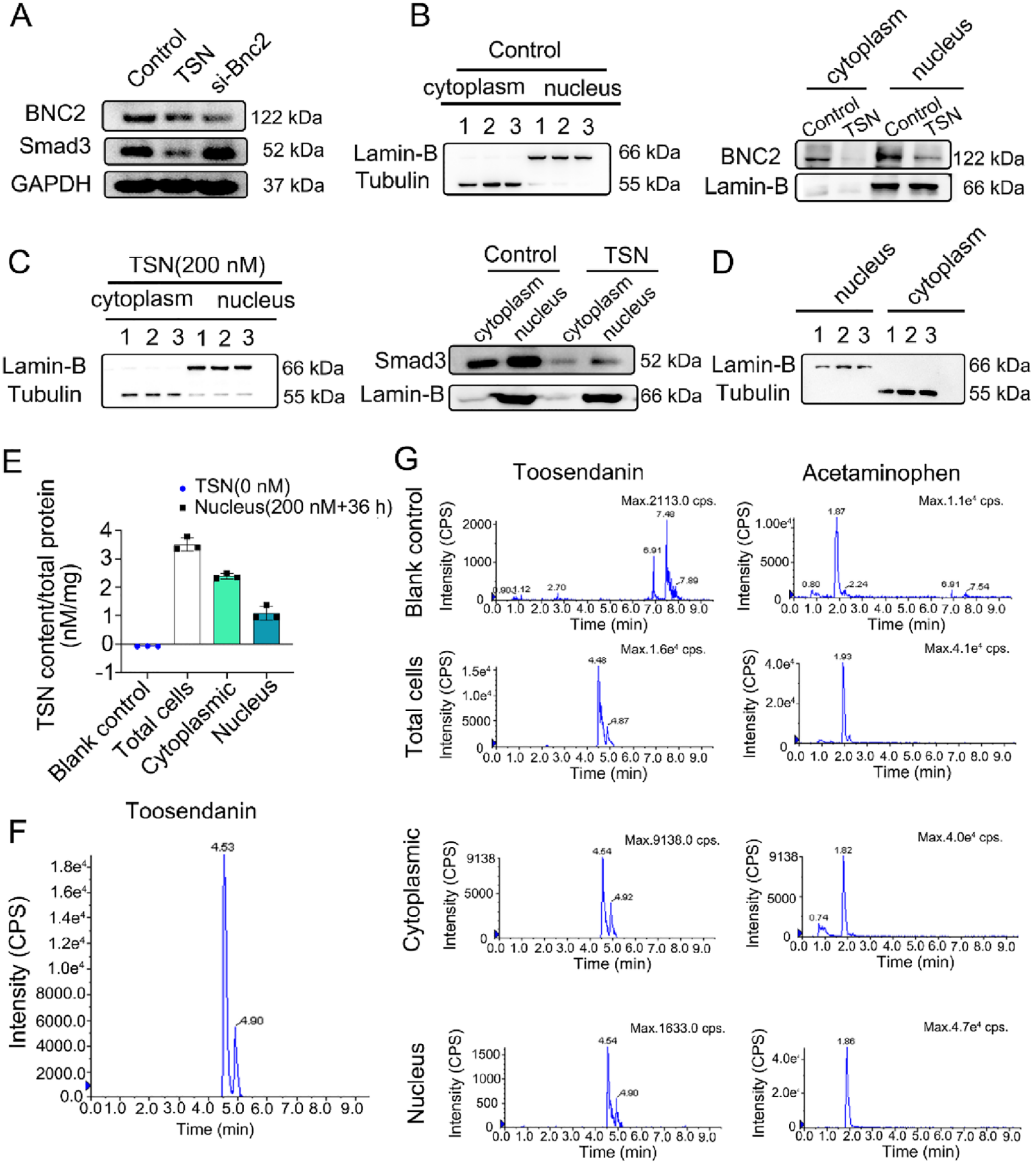
HPLC‐MS/MS analysis showed that TSN could enter the cytoplasm and nucleus to downregulate BNC2 and Smad3. (A) Western blot analysis of BNC2 and Smad3 protein expression in B16‐F10 cells after TSN treatment (200 nM) or si‐BNC2 transfection for 36 h. (B—C) Western blot analysis of Lamin B (nuclear marker), Tubulin (cytoplasmic marker), BNC2, and Smad3 expression in nuclear and cytoplasmic fractions isolated from control and TSN‐treated cells. (D) Western blot analysis of Lamin B and Tubulin confirming the efficiency of nuclear and cytoplasmic fractionation in control and TSN‐treated cells. (E—G) Quantification of TSN levels in cytoplasmic and nuclear fractions of cells treated with TSN (200 nM) for 36 h, as determined by HPLC–MS/MS. n = 3. The differences were considered statistically significant with *p < 0.05, ^**^ *p* < 0.01, ^***^ *p* < 0.001 and ^****^ *p* < 0.0001.

As shown above, BNC2 plays a critical role in melanoma progression and metastasis; TSN suppresses BNC2 expression, likely through direct binding to BNC2. To understand the mechanism, we noted that previous studies have demonstrated that BNC2 can interact with Smad3 to form a transcriptional complex that regulates its own expression as well as downstream genes [16]. In this study, we observed that TSN treatment for 36 h resulted in a marked decrease in both BNC2 and Smad3 protein levels (Figure 7A). In contrast, knockdown of BNC2 did not affect Smad3 expression (Figure 7A). This finding raised the possibility that TSN may promote the degradation of the BNC2‐Smad3 complex in the nucleus. To test this hypothesis, we performed nuclear and cytoplasmic fractionation followed by analysis of BNC2 expression after TSN treatment (Figure 7B,C). Interestingly, in addition to its predominant nuclear localization, BNC2 was also detected in the cytoplasm (Figure 7B). Further, TSN treatment markedly reduced BNC2 and Smad3 protein levels in both nuclear and cytoplasmic fractions (Figure 7B,C). These findings indicate that TSN is capable of inducing BNC2 degradation not only in the nucleus but also in the cytoplasm.

To further confirm that TSN enters both the cytoplasm and nucleus to exert its function, the cytoplasmic and nuclear fractions of TSN‐treated cells were isolated (Figure 7D), and the intracellular TSN levels were quantified using HPLC‐MS/MS (Figure 7E—G). The analysis confirmed that TSN was present in both the cytoplasm and nucleus, although its abundance in the nucleus was significantly lower than that in the cytoplasm (Figure 7E). In summary, our findings demonstrate that TSN can enter cells to induce BNC2 and Smad3 degradation in both the cytoplasm and nucleus.

### 3.8 TSN Promotes BNC2 Ubiquitination and Degradation via CRBN

**FIGURE 8:**
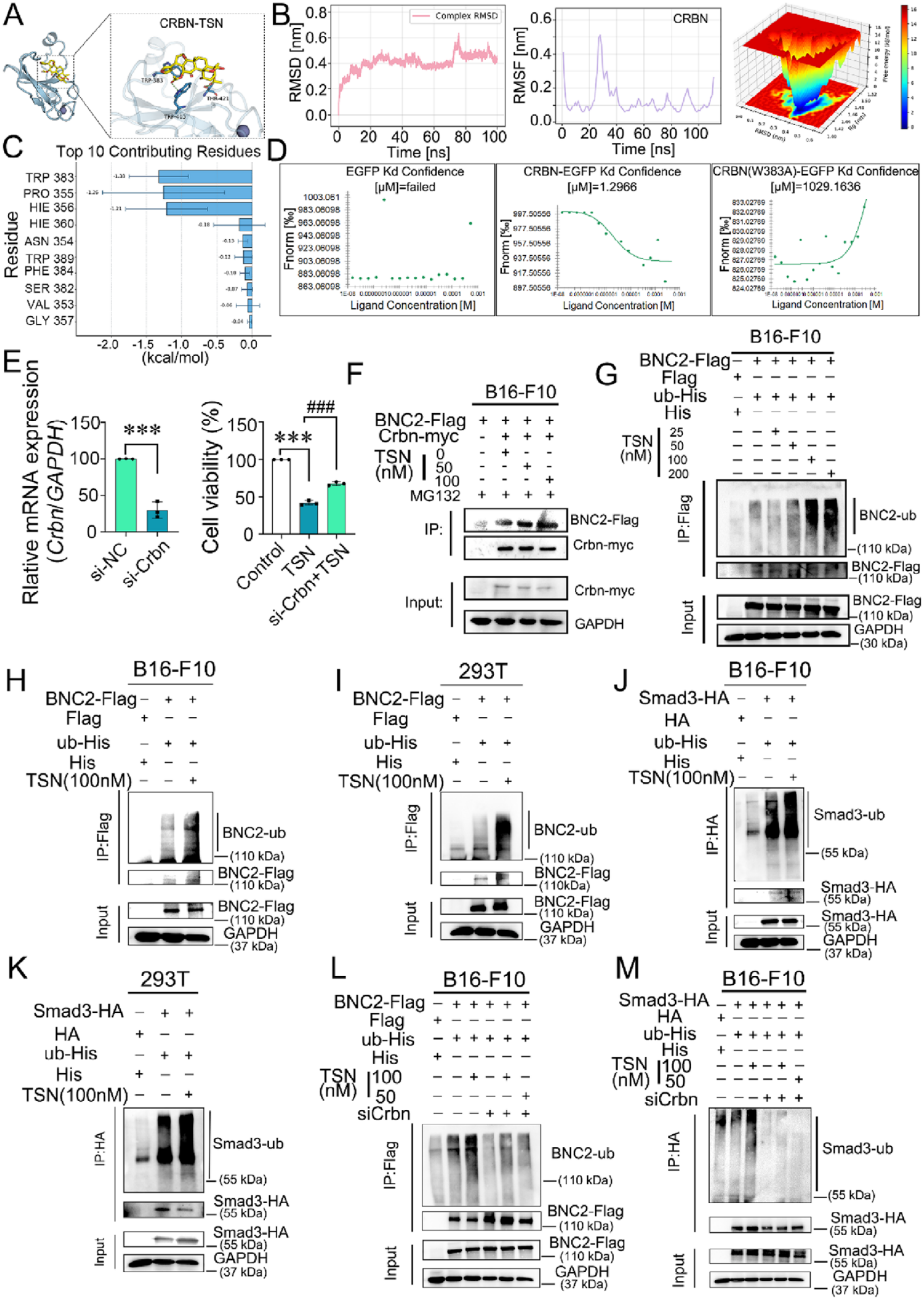
TSN promotes CRBN‐dependent ubiquitination and degradation of BNC2 and Smad3. (A) Based on the binding mode of Crbn and TSN obtained from docking, the left panel shows an overall view, and the right panel shows a close‐up view. (B) Complex RMSD in MD, Protein RMSF in MD, and FEL result. (C) Binding energy contributions of key amino acid residues (D) Representative images of microscale thermophoresis (MST) in the EGFP, CRBN‐EGFP, and CRBN(W383A)‐EGFP groups. (E) qPCR analysis of *Crbn* knockdown efficiency following si‐Crbn transfection and MTT assay assessing the cell viability of the TSN group and TSN‐treated cells after *Crbn* knockdown, n = 3. (F) Co‐immunoprecipitation analysis of the interaction between BNC2 and Crbn in B16‐F10 cells transfected with pSL4‐BNC2‐Flag and Crbn‐Myc plasmids and treated with increasing concentrations of TSN (0, 50, and 100 nM) for 36 h. (G) Analysis of BNC2 ubiquitination in B16‐F10 cells transfected with pSL4‐BNC2‐Flag and ub‐His plasmids and treated with TSN (25, 50, 100, and 200 nM) for 36 h. (H—I) Ubiquitination and protein level analysis of BNC2 in B16‐F10 and 293T cells transfected with pSL4‐BNC2‐Flag and ub‐His plasmids and treated with TSN (100 nM) for 36 h. (J—K) Ubiquitination and protein level analysis of Smad3 in B16‐F10 and 293T cells transfected with Smad3‐HA and ub‐His plasmids and treated with TSN (100 nM) for 36 h. (L—M) Analysis of BNC2 and Smad3 ubiquitination in B16‐F10 cells co‐transfected with pSL4‐BNC2‐Flag, Smad3‐HA, ub‐His plasmids, and si‐Crbn, followed by TSN treatment (50 and 100 nM) for 36 h. Statistical significance was defined as ^***^ *p* < 0.001. ^###^ *p* < 0.001.

Since zinc finger protein type transcription factor degradation typically occurs through a ubiquitin‐mediated pathway [32], we hypothesized that TSN may facilitate the ubiquitin‐dependent degradation of BNC2. Thalidomide and its derivatives target cereblon (CRBN) to mediate the degradation of zinc finger proteins [33, 34, 35]. Recently, thalidomide has also been identified as a compound promoting CRBN‐mediated ubiquitination of BNC2 [16]. Molecular docking revealed that TSN binds to the Crbn protein with a binding affinity score of −6.04 kcal/mol (Figure 8A). Molecular dynamics simulations confirmed that the complex maintained conformational stability after an initial relaxation period. The free energy landscape (FEL) exhibited a single deep energy well, indicating high conformational stability throughout the simulation (Figure 8B). Per‐residue binding energy decomposition identified TRP383 (−1.33 kcal/mol), PRO355 (−1.26 kcal/mol), and HIE356 (−1.21 kcal/mol) as the dominant contributors among the key residues driving ligand binding, underscoring their decisive role in system stability (Figure 8C). MST experiments further confirmed that mutation of the TRP383 residue reduced the binding affinity from 1.2966 to 1029.1636 µM, validating the TSN‐Crbn interaction (Figure 8D).

To determine whether this interaction confers a thalidomide‐like mechanism of action, we knocked down Crbn expression in B16‐F10 cells and observed that Crbn silencing significantly reversed the TSN‐induced decrease in cell viability (Figure 8E). Co‐immunoprecipitation assays revealed that TSN dose‐dependently promoted the interaction between BNC2 and Crbn (Figure 8F). We treated B16‐F10 cells with increasing concentrations of TSN and observed a dose‐dependent enhancement of BNC2 ubiquitination (Figure 8G). Consistently, TSN markedly induced BNC2 ubiquitination in both B16‐F10 and 293T cells (Figure 8H,I). Notably, TSN also significantly promoted the ubiquitination and degradation of Smad3 (Figure 8J,K). Crbn knockdown effectively reversed TSN‐induced ubiquitination of both BNC2 and Smad3 (Figure 8L,M).

Collectively, these findings demonstrate that TSN binds to both CRBN and BNC2, recruiting CRBN to BNC2 and thereby inducing ubiquitin‐mediated degradation of the BNC2‐SMAD3 complex.

### 3.9 TSN Nanoliposomes Significantly Enhance TSN‐Mediated Inhibition of Melanoma Proliferation and Metastasis

**FIGURE 9:**
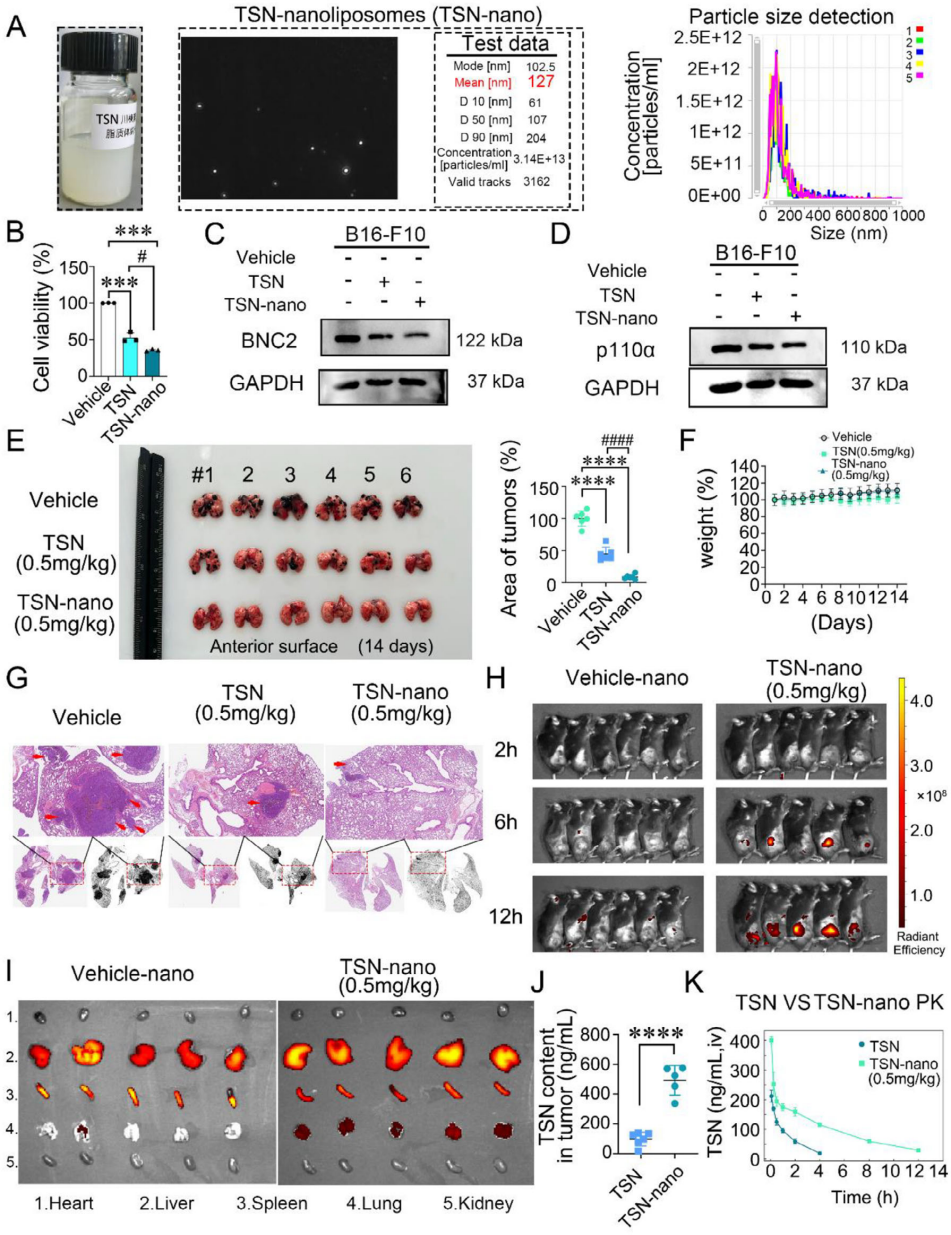
TSN nanoliposomes enhance the anti‐metastatic efficacy of TSN. (A) Preparation of TSN nanoliposomes by the thin‐film dispersion method and analysis of particle size distribution and dispersion characteristics using a Malvern Panalytical particle size analyzer. n = 5. (B) MTT assay assessing the viability of B16‐F10 cells treated with TSN (200 nM) or TSN nanoliposomes (200 nM). n = 3. (C—D) Western blot analysis of BNC2 and p110α protein expression in cells treated with TSN (200 nM) or TSN nanoliposomes (200 nM) for 36 h. (E) Representative images of lungs from a melanoma lung metastasis mouse model following treatment with TSN (0.5 mg/kg) or TSN nanoliposomes (0.5 mg/kg), together with quantification of metastatic lung lesion areas. n = 6. (F) Body weight monitoring of mice in the melanoma lung metastasis model following intraperitoneal injection of TSN or TSN nanoliposomes. (G) Representative H&E‐stained lung sections from mice treated with free TSN or TSN nanoliposomes. Scale bar: 1000 and 200 µm. (H) Mice were injected via the tail vein with liposomes or TSN‐liposomes (2 mg/kg), and tumor fluorescence intensity was measured by in vivo imaging at 2, 6, and 12 h after injection. (I) At the experimental endpoint, different organs were harvested from mice and subjected to ex vivo imaging. (J) The concentration of TSN in tumor tissues was measured by HPLC‑MS. (K) Pharmacokinetic results (plasma concentration‑time curves) of TSN and TSN nanoliposomes. Statistical significance was defined as ^*^ *p* < 0.05, ^**^ *p* < 0.01, ^***^ *p* < 0.001 and ^****^ *p* < 0.0001. ^##^ < 0.01, ^###^ *p* < 0.001 and ^####^ *p* < 0.0001.

Although TSN exerts significant anti‐melanoma effects by promoting BNC2 degradation, the cellular uptake of TSN was relatively low during our study. Increasing TSN dosage may lead to uncontrollable hepatotoxicity and neurotoxicity [36]. To solve this problem, we therefore utilized to take advantage of nanoliposomal encapsulation technology to enhance the efficacy of TSN while circumventing potential side‐effects [37]. The detailed TSN nanoliposome formulation is shown in Figure S6A. The TSN nanoliposomes appeared as a light milky‐white suspension. The dispersion state and particle size distribution of TSN nanoliposomes were analyzed using a Malvern Panalytical system, revealing uniform dispersion without aggregation in real‐time imaging (Figure 9A and Video S1). The average particle size of TSN nanoliposomes was 127 nm (Figure 9A and Figure S6B), with a drug encapsulation efficiency of 55.26% (Figure S6C,D). TSN nanoliposomes exhibited significantly greater inhibition of cell proliferation compared with free TSN at the same concentration (Figure 9B). TSN nanoliposomes induced more pronounced degradation of BNC2 protein and p110α protein than TSN (Figure 9C,D). In a lung metastasis model, TSN nanoliposomes also exhibited superior inhibition of metastasis compared to TSN (Figure 9E and Figure S6E,F), without causing significant body weight loss in mice (Figure 9F). H&E staining further revealed that both the number and area of metastatic lung foci were markedly reduced in the TSN nanoliposome group compared with the TSN group (Figure 9G and Figure S6G). Metastasis‐related markers were further evaluated by IHC. TSN nanoliposome treatment significantly decreased the protein expression of BNC2, Ki‐67, p110α, Vimentin, and N‐cadherin, while increasing the expression of ZO‐1 in tumor tissues (Figure S6H). H&E staining of the liver, brain, and kidneys from treated mice revealed no detectable toxicity from either TSN or TSN nanoliposomes, indicating that the administered dose of 0.5 mg/kg was relatively safe (Figure S7A–D). No significant elevation in serum ALT or AST levels was detected, further supporting the safety of TSN and TSN nanoliposomes administration (Figure S7E).

To further evaluate the tumor‐targeting capacity of TSN nanoliposomes, we performed in vivo imaging and observed substantial accumulation of TSN nanoliposomes in tumor tissue at 12 h, whereas the control nanobody group exhibited no detectable tumor targeting (Figure 9H and Figure S8A). Ex vivo imaging of major organs at the experimental endpoint revealed that nanoliposomes predominantly accumulated in the liver and kidneys, whereas TSN encapsulated in nanobodies displayed a lung‐targeting profile (Figure 9I and Figure S8A). Quantification of TSN levels in tumor tissues showed that TSN nanoliposomes achieved significantly higher tumor accumulation compared with free TSN (Figure 9J and Figure S8B). These results demonstrate that TSN nanoliposomes exhibited significantly enhanced tumor targeting.

Finally, pharmacokinetic analysis of TSN was conducted. Free TSN and TSN nanoliposomes both reached peak concentration at 5 min post‐injection via tail vein administration (Figure 9K and Figure S8C). The C_max_ of LIP (401.04 ng/mL) was 1.89‐fold higher than that of free TSN (212.13 ng/mL). Liposomal encapsulation significantly prolonged the elimination half‐life from 1.29 to 4.10 h (3.18‐fold increase). The AUC_0_−_∞_ was elevated from 314.53 ng·h/mL to 1344.15 ng·h/mL, yielding a relative bioavailability of 427.35%, and the MRT_0_−_∞_ was extended from 1.79 to 5.77 h (3.22‐fold) (Figures S8C and S9A). These results demonstrate that liposomal encapsulation significantly improves the pharmacokinetic profile of TSN, prolongs systemic circulation time, and enhances systemic exposure, indicating its potential for improved therapeutic efficacy.

### 3.10 TSN Combined with the Clinical Drug Dacarbazine Significantly Treats Melanoma Lung Metastasis

Anti‐PD‐1/PD‐L1 immune checkpoint inhibitors (such as nivolumab and pembrolizumab) have become one of the first‐line standard treatment options for metastatic melanoma, with durable responses achievable in a subset of patients [38]. However, over 40% of patients fail to respond to immunotherapy or develop acquired resistance, and the benefits are even more limited in patients with BRAF wild‐type tumors and those with visceral metastases such as lung metastases. In this context, dacarbazine (DTIC) remains an irreplaceable first‐line chemotherapeutic agent, widely used in patients who have failed immunotherapy and in those with advanced metastatic disease [39, 40]. However, its single‐agent objective response rate is only 10%–20%, with a median overall survival of 5.6–9.4 months, rendering its efficacy far from satisfactory [41]. Dacarbazine‐based combination therapy increased the objective response rate by approximately 60% (RR = 1.60, 95% CI: 1.27–2.01) and improved the 1‐year survival rate by approximately 26% (RR = 1.26, 95% CI: 1.14‐1.39) compared with DTIC alone, indicating a clear advantage of combination therapy in enhancing DTIC response rate and survival benefit. Therefore, identifying combination strategies that can sensitize DTIC holds important clinical significance for improving the prognosis of metastatic melanoma, particularly in patients with lung metastasis.

**FIGURE 10:**
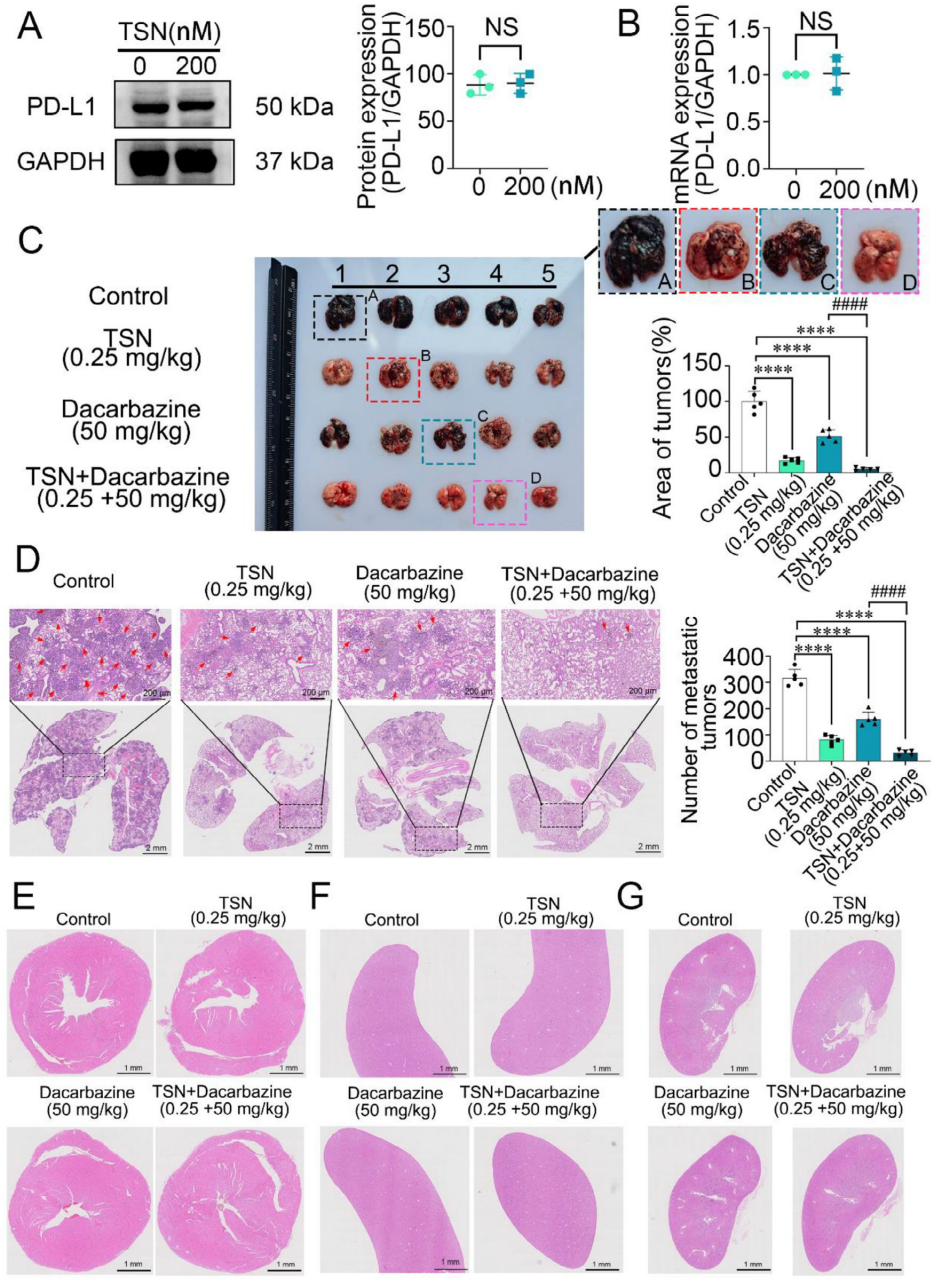
TSN enhances the inhibitory effect of dacarbazine on melanoma lung metastasis. (A,B) Western blot and RT‐qPCR analysis of PD‐L1 protein and mRNA expression in melanoma cells treated with TSN. (C) Quantification of lung metastatic foci in mice from the control, TSN, dacarbazine, and TSN + dacarbazine groups. (D) Representative H&E staining images of lung tissues from each group. Scale bar:2 mm (E–G) H&E staining of heart (E), liver (F), and kidney (G) tissues assessing systemic toxicity. Scale bar:1 mm. Statistical significance was defined as ^***^ *p* < 0.001 and ^****^ *p* < 0.0001. ^####^ *p* < 0.0001.

Experimental results showed that TSN had no significant effect on either the protein or mRNA expression of PD‐L1 in melanoma cells (Figure 10A,B). However, compared with the control group, the combination of TSN and dacarbazine exhibited a significant synergy in suppressing melanoma lung metastasis. Notably, TSN treatment alone also displayed superior anti‐metastasis efficacy compared with that of dacarbazine monotherapy (Figure 10C). H&E staining of lung tissue further confirmed that the combination group exhibited fewer metastatic foci with smaller lesion areas (Figure 10D). Toxicity evaluation revealed that neither the combination of TSN and dacarbazine nor the individual monotherapy groups caused significant toxic damage to the heart (Figure 10E), liver (Figure 10F), and kidneys (Figure 10G). These findings indicate that TSN significantly enhances the inhibitory effect of dacarbazine on melanoma lung metastasis.

## 4 Discussion

BNC2 has been reported to exert opposing functions across different cancer types: it acts as a tumor suppressor in ovarian cancer through its involvement in DNA damage repair and transcriptional repression, yet shows oncogenic associations in breast, lung, and prostate cancers [12, 13, 14, 15]. The present study addresses this apparent paradox in the context of melanoma. Our data demonstrate that BNC2 is markedly overexpressed in melanoma, with the highest expression levels observed in metastatic lesions, and that elevated BNC2 expression correlates with poor patient prognosis. Through in vitro cellular assays and an in vivo B16‐F10 lung metastasis model, combined with BNC2 overexpression and knockdown manipulations, we establish that BNC2 is a critical driver of melanoma cell proliferation and metastasis. Mechanistically, BNC2 functions as a transcriptional activator of PIK3CA, thereby engaging the PI3K signaling pathway. These findings identify BNC2 as an oncogenic driver in melanoma, highlighting its context‐dependent role and enhancing the understanding of its function.

Our further investigation identified TSN as a small‐molecule inhibitor that targets BNC2 for degradation. TSN directly binds to BNC2 and recruits the E3 ubiquitin ligase CRBN, promoting CRBN‐dependent ubiquitination and degradation of BNC2. Notably, TSN‐mediated BNC2 degradation also leads to concomitant degradation of Smad3, a BNC2‐interacting transcription factor that promotes tumor proliferation and metastasis. Thus, TSN simultaneously disrupts two oncogenic regulators, providing a previously uncovered molecular basis for its robust anti‐melanoma activity.

Given the limited membrane permeability of TSN and the risk of hepatotoxicity and neurotoxicity at higher doses, we developed TSN nanoliposomes. This formulation enhanced cellular uptake, improved tumor accumulation, and prolonged systemic circulation, resulting in superior inhibition of melanoma growth and metastasis compared with free TSN. The combination of TSN with dacarbazine further enhanced anti‐metastatic efficacy, highlighting a potential strategy for sensitizing melanoma to conventional chemotherapy. TSN nanoliposomes were designed to overcome the key translational barriers of free TSN‐poor cellular uptake, narrow therapeutic window, and dose‐dependent hepatotoxicity and neurotoxicity. Compared with other nanodelivery platforms (polymeric, inorganic, or targeted nanoparticles), our liposomal formulation offers a favorable balance of efficacy, safety, tumor targeting, pharmacokinetics, and manufacturing simplicity. The platform achieves prolonged circulation (half‐life extended from 1.29 to 4.10 h, relative bioavailability 427%), pronounced tumor accumulation, superior antitumor efficacy (including suppression of pulmonary metastasis), and a clean safety profile at 0.5 mg/kg. These features position TSN nanoliposomes as a practical and scalable candidate for integration with existing melanoma therapies, including chemotherapy, targeted therapy, and immunotherapy. Further combination studies and exploration of next‐generation nanocarriers are warranted.

**FIGURE 11:**
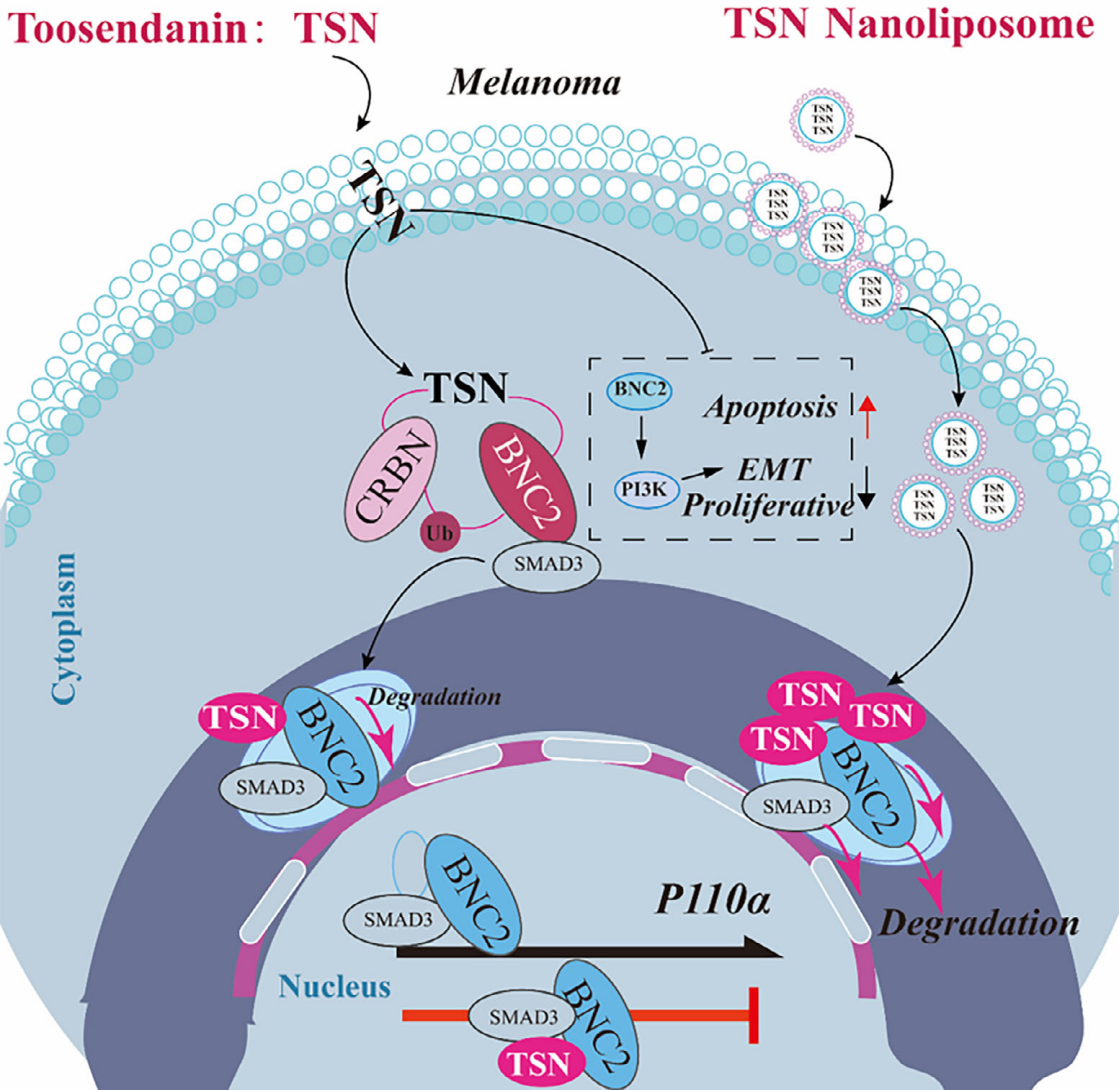
Mechanism diagram of TSN inhibiting melanoma proliferation and metastasis.

In summary, these findings establish BNC2 as a context‐dependent driver of melanoma proliferation and metastasis, and identify TSN as a natural small‐molecule degrader of BNC2 that acts through CRBN‐dependent ubiquitination (Figure 11). TSN nanoliposomes further enhance its therapeutic potential, positioning this strategy as a promising approach for BNC2‐driven malignancies, including melanoma.

## Author Contributions

**Hui Dai**: article design, literature review, in vivo and in vitro experiments, data processing, article writing. **Xin Mei Zhang**: clinical sample collection, experimental protocol review guidance, experimental support. **Jing Cheng Zhang**: in vitro and in vivo experiments. **Xu Ting Deng**: in vitro and in vivo experiments. **Qiao Liu**: experiments related to mass spectrometry analysis. **Wu Yin** & **Fang Zhou Yin**: writing – editing, article design, writing – original draft, supervise experiments, software, project administration, methodology, investigation, funding acquisition, formal analysis, data curation.

## Conflicts of Interest

The authors declare no conflicts of interest.

## Supporting information




**Supporting File 1**: advs77458‐sup‐0001‐SuppMat.docx.

**Supporting File 2**: advs77458‐sup‐0002‐TableS1.xlsx.

**Supporting File 3**: advs77458‐sup‐0003‐TableS2.xlsx.

**Supporting File 4**: advs77458‐sup‐0004‐Video1.avi.

## Data Availability

Research data are not shared.
