# Supplementary material for: Natural Product Toosendanin Suppresses the Malignant Development of Skin Melanoma by Targeting BNC2 for Degradation
Source: Adv Sci (Weinh). 2026 Aug 30:e77458. Online ahead of print. doi: 10.1002/advs.77458 (PMC13526533; doi:10.1002/advs.77458)
Supplement: Supplementary file 1 [file ADVS-9999-e77458-s002.docx]

**Supplementary data**

**Figure S1.** **Statistics on lung metastasis area and observation of liver and kidney metastases in melanoma-bearing mice.** (A) Representative images of resected subcutaneous tumors from C57BL/6 mice treated with TSN (0.0 mg/kg and 0.5 mg/kg), n = 3. (B) Quantification of the residual wound area in the scratch assay, n = 3. (C) Representative images and quantitative analysis of metastatic tumor areas on the posterior surface of the lungs. (D) Images of metastatic lesions on the anterior and posterior lung surfaces processed and quantified using ImageJ software. (E) ImageJ-processed images showing metastatic lesions on the anterior lung surface in mice bearing highly metastatic B16-F10 melanoma. (F) Representative images of liver tissues from mice in the B16-F10 melanoma lung metastasis model following TSN treatment. (G) Representative images of kidney tissues from mice in the B16-F10 melanoma lung metastasis model following TSN treatment. (H) Representative images of liver tissues from mice in the highly metastatic B16-F10 melanoma lung metastasis model following TSN treatment. (I) Representative images of kidney tissues from mice in the highly metastatic B16-F10 melanoma lung metastasis model following TSN treatment.

**Figure S2.** **Representative IHC staining images after TSN treatment in a mouse melanoma lung metastasis model.** (A) Representative immunohistochemical staining images showing the expression of Ki-67, E-cadherin, N-cadherin, Vimentin, Occludin, and ZO-1 in lung metastatic lesions from mice treated with TSN. (B) Representative immunohistochemical staining images showing the expression of S-100 in lung metastatic lesions from mice treated with TSN.

**Figure S3.** **Effect of high expression of BNC2 on survival curves of different tumors.** (A) Analysis of BNC2 expression across different cell types in normal human skin tissue based on the Human Protein Atlas single-cell database. (B-F) Overall survival (OS) analys**es** of patients with pancreatic adenocarcinoma (PAAD), lower-grade glioma (LGG), glioblastoma multiforme (GBM), breast carcinoma (BRCA), and bladder carcinoma (BLCA) using the GEPIA database. (G-H) Representative hematoxylin and eosin (H&E) staining and BNC2 immunohistochemical staining of tumor tissue sections from patients with skin cutaneous melanoma (SKCM). Scale bar: 2 mm and 200 μm. Clinical sample collection was approved by the hospital ethics committee. SKCM patients. Scale bar, 2mm and 200 μm. Clinical samples were approved by the hospital ethics committee.

**Figure S4. TSN targets BNC2 to inhibit the expression of BNC2 and PIK3CA.** (A) KEGG pathway **enrichment analysis** showing protein pathways associated with BNC2 expression in patients with skin cutaneous melanoma (SKCM) based on the TCGA database. (B) Correlation analysis between BNC2 and PIK3CA expression in SKCM using the TCGA database. (C) mRNA expression levels of BNC2 in normal skin tissue, primary SKCM, and metastatic SKCM analyzed using the GEO dataset GSE15605. (D) Single-cell transcriptomic analysis of melanoma tissues showing the expression of BNC2 and PIK3CA across different cell populations, based on data from the Single Cell Portal database. (E) Representative immunohistochemical staining images showing p110α expression in melanoma patient tissue sections. Scale bar: 50 μm.

**Figure S5.** **Validation of BNC2 knockdown or overexpression efficiency.** (A-C) qPCR analysis confirming the transfection efficiency of si-BNC2, sh-BNC2, and pSL4-BNC2. (D) mCherry fluorescence imaging verifying the successful establishment of BNC2 knockdown stable cell lines. Scale bar: 200 μm. (E) Western blot analysis of BNC2 protein expression in polyclonal stable BNC2 knockdown cell lines. (F) ImageJ-processed images showing metastatic tumor areas on the anterior surface of the lungs. (G) Representative immunohistochemical staining showing the expression of BNC2, Ki-67, Vimentin, E-cadherin, and N-cadherin in the sh-NC and sh-BNC2 groups of the mouse melanoma lung metastasis model. Scale bar: 50 μm. (H) Representative images of liver tissues from mice in the B16-F10 melanoma lung metastasis model. (I) Representative images of kidney tissues from mice in the B16-F10 melanoma lung metastasis model.

**Figure.S6.** **TSN-nanoliposomes inhibit melanoma metastasis.** (A) Detailed formulation of TSN nanoliposomes. (B) Comparison of particle size distributions between TSN nanoliposomes and blank nanoliposomes analyzed using a Malvern Panalytical particle size analyzer. (C-D) Determination of TSN encapsulation efficiency in nanoliposomes by HPLC–MS following ultrafiltration centrifugation. *Standard TSN* indicates the TSN reference standard, and *Free TSN* indicates non-encapsulated TSN. (E) Representative images showing metastatic tumor lesions on the anterior surface of the lungs. (F) Representative images showing metastatic tumor areas on the dorsal surface of the lungs. (G) Quantification of the number of metastatic nodules in lung tissues, n = 3. (H) Representative IHC staining statistical results of BNC2, p110α, Ki67, N-cadherin, Vimentin and ZO-1 expression in the mouse melanoma lung metastasis model after TSN and TSN nanoliposomes treatment, n = 6. Statistical significance was defined as *P < 0.05, **p < 0.01, ***P < 0.001 and ****P < 0.0001. ## < 0.01, ###P < 0.001 and ####P < 0.0001.

**Figure S7. Toxicity evaluation of TSN and TSN nanoliposomes.** (A-D) Representative H&E-stained sections of the liver, brain, and kidneys from mice treated with TSN or TSN nanoliposomes (0.5 mg/kg). No apparent histopathological abnormalities were observed compared with the control group. Scale bar, 1 mm. (E) Serum ALT and AST levels (U/L) in mice after treatment with TSN or TSN nanoliposomes. (0.5 mg/kg). Statistical significance was defined as ^NS^P > 0.05.

**Figure S8. Pharmacokinetic profiles of TSN and TSN nanoliposomes.** (A) Mice were injected via the tail vein with liposomes or TSN liposomes (2 mg/kg), and tumor fluorescence intensity was measured by in vivo imaging at 2, 6, and 12 hours after injection. (B) Representative HPLC-MS chromatograms of TSN in tumor tissues. (C) Detailed pharmacokinetic parameters of TSN and TSN nanoliposomes.

**Figure S9. HPLC-MS quantification of TSN in plasma.** (A) Representative HPLC‑MS chromatograms of TSN in plasma at different time points.
